# Mild hypothermia upregulates *myc* and *xbp1s* expression and improves anti-TNFα production in CHO cells

**DOI:** 10.1371/journal.pone.0194510

**Published:** 2018-03-22

**Authors:** Mauro Torres, Roberto Zúñiga, Matias Gutierrez, Mauricio Vergara, Norberto Collazo, Juan Reyes, Julio Berrios, Juan Carlos Aguillon, Maria Carmen Molina, Claudia Altamirano

**Affiliations:** 1 Escuela de Ingeniería Bioquímica, Pontificia Universidad Católica de Valparaíso, Valparaíso, Chile; 2 Centro de InmunoBiotecnología, Programa D. de Inmunología, Instituto de Ciencias Biomédica (ICBM), Facultad de Medicina, Universidad de Chile, Santiago, Chile; 3 Doctorado en Química, Universidad República Oriental del Uruguay, Montevideo, Uruguay; 4 Instituto de Química, Pontificia Universidad Católica de Valparaíso, Valparaiso, Chile; 5 CREAS CONICYT Regional GORE, Valparaiso, Chile; Ecole Polytechnique, CANADA

## Abstract

Chinese hamster ovary (CHO) cells are the most frequently used host for commercial production of therapeutic proteins. However, their low protein productivity in culture is the main hurdle to overcome. Mild hypothermia has been established as an effective strategy to enhance protein specific productivity, although the causes of such improvement still remain unclear. The self-regulation of global transcriptional regulatory factors, such as Myc and XBP1s, seems to be involved in increased the recombinant protein production at low temperature. This study evaluated the impact of low temperature in CHO cell cultures on *myc* and *xbp1s* expression and their effects on culture performance and cell metabolism. Two anti-TNFα producing CHO cell lines were selected considering two distinct phenotypes: i.e. maximum cell growth, (CN1) and maximum specific anti-TNFα production (CN2), and cultured at 37, 33 and 31°C in a batch system. Low temperature led to an increase in the cell viability, the expression of the recombinant *anti-TNFα* and the production of anti-TNFα both in CN1 and CN2. The higher production of anti-TNFα in CN2 was mainly associated with the large expression of *anti-TNFα*. Under mild hypothermia *myc* and *xbp1s* expression levels were directly correlated to the maximal viable cell density and the specific anti-TNFα productivity, respectively. Moreover, cells showed a simultaneous metabolic shift from production to consumption of lactate and from consumption to production of glutamine, which were exacerbated by reducing culture temperature and coincided with the increased anti-TNFα production. Our current results provide new insights of the regulation of *myc* and *xbp1s* in CHO cells at low temperature, and suggest that the presence and magnitude of the metabolic shift might be a relevant metabolic marker of productive cell line.

## Introduction

Over the years, the demand for recombinant proteins as biopharmaceuticals has increased dramatically, attaching a special relevance to monoclonal antibody production [1]. Since these macromolecules are the keystones for the development of new treatments facing more effectively diseases such as long-term autoimmune disorders or some cancers [2–5], they are becoming very important in the biopharmaceutical market. Proof of that are their positive clinical results and increased approval of therapeutic antibody drugs for clinical uses by international organisations in the United States and Europe [1]. Such scenario of increased demand for these therapeutic agents therefore places considerable pressure on the development of highly efficient production processes to develop less expensive drugs [6,7].

To this date, Chinese hamster ovary (CHO) cells are the main platform for the production of a great number of recombinant therapeutic antibodies [8] due to their easy gene manipulation, adaptation to suspension cultures and capacity to properly perform post-translational modification, particularly glycosylations [9,10]. The vast majority of anti-TNFα drugs are produced by recombinant CHO cells [6,7]. However, the principal hurdle for these cell lines to overcome is the low productivity of recombinant proteins reached by these production processes [11]. Since production of a recombinant protein is directly related to specific productivity and the integral of viable cell (IVC), efforts to maximize production are directed towards a synergistic combination of both approaches selecting highly productive cell lines and optimizing environmental culture condition.

One strategy for significantly enhancing specific productivity in CHO cell culture is the application of mild hypothermia, either by temperature down-shift [12–17] or by low temperature acclimatization [18,19]. A low temperature, a few degrees below 37°C (usually from 35°C to 30°C), enables an increase in the production of a recombinant protein, with no significant changes in its quality (*in vivo* biological activity)[10]. In batch cultures, hypothermic growth leads to a series of changes at the physiological level, improving cell viability and culture longevity, while decreasing cell density, specific growth rate and protease activity [14–16,18,20–22]. Along with this, cell metabolism is widely affected by reduced culture temperature, registering an overall decrease in the utilization of carbon and energy sources [22,23] and the generation of by-products (e.g. lactate) [15,24], accompanied by cell cycle arrest in G1 [21,25], an improvement in the transcription and stability of foreign genes [16,21,26] as well as an increase in the folding and processing capacity of endoplasmic reticulum (ER) [27,28].

The improvements in protein production under mild hypothermic conditions to some extent are associated with expression levels of global transcriptional regulatory factors involved in either carbon metabolism or endoplasmic reticulum protein processing [29]. On one hand, Myc regulates cell growth and proliferation stimulating the expression of many genes involved in glucose uptake, glycolysis, glutaminolysis and the fate of glycolytic pyruvate [30]. Previous studies have shown that the overexpression of *myc* in CHO cells increases the specific growth rate [31] without negatively affecting the specific protein productivity [32]. Moreover, we recently investigated the effect of reducing culture temperature on the transcriptomics of CHO cells. In that work, we evidenced that CHO cells under mild hypothermia upregulates *myc*, improves specific hr-tPA productivity and decreases significantly lactate production compared to the control (37°C) [29]. These observations have been also reported in the CHO cell line producing erythropoietin, where the improved specific erythropoietin productivity at low culture temperatures was in part associated with the upregulated expression of *myc* [26]. Although other transcriptional regulatory factors involved in cell proliferation and carbon metabolism pathways that might also be affected by the temperature reduction either at the same level of *myc* or downstream [29], the lack of evidence regarding their effect on the culture performance led us to focus our efforts exclusively on *myc*. On the other hand, XBP1s regulates protein secretion and the unfolded protein response, acting as a transcriptional activator inducing key genes associated with protein secretion and increased biosynthesis within the endoplasmic reticulum (ER) [33]. Different authors agree on the fact that the limiting steps in the protein production in mammalian cells occur at translational or post-translational levels, particularly in the protein processing steps in the ER of cells [34,35]. However, attempts to overexpress XBP1s in CHO cells have had dissimilar results. In some cases, engineered CHO cells overexpressing XBP1s have enabled to significantly enhance protein productivity [36–38] due to an improvement of the secretory capacity of cells [39], while in another cases there were not any improvement of recombinant protein production [40–42]. Our previous study additionally demonstrated that CHO cells at low culture temperature highly upregulate different ER chaperones and other folding and trafficking associated proteins at the most productive condition [29]. Therefore, the effect of XBP1s on protein production could depend on the protein traffic level of cells. In this context, we proposed that the application of mild hypothermic conditions to CHO cells producing recombinant anti-TNFα fosters endogenous changes in expression levels of *myc* and *xbp1s* that may lead to increased volumetric productivity.

In the present work, we aimed to evaluate the effect of mild hypothermia on the culture performance of two highly productive CHO cell lines producing anti-TNFα, the impact of culture temperature on *myc* and *xbp1s* expression, and their relationship with cell metabolism and anti-TNFα productivity. To do so, we successfully isolated two clonally derived CHO cells producing anti-TNFα based on the maximum cell growth (CN1) and maximum protein production (CN2) criteria. With these two CHO cell lines, we seek to represent two different phenotypes in which a higher expression of *myc* in CN1 and *xbp1s* in CN2 are expected both physiological and low temperatures. For both clones, we evaluated the effect of low culture temperature on cell growth, recombinant protein production, cell metabolism, and expression levels of myc, xbp1s and anti-TNFα.

## Material and methods

### Cell culture and media

Suspension-adapted CHO DG44 cells, DHFR-deficient, were obtained from ThermoFisher Scientific (USA) and initially cultured in a chemically defined CD-DG44 medium (Gibco, USA) with a mixture of sodium hypoxanthine and thymidine (HT). Transfections were carried out using FreeStyle™ MAX System Reagent (Invitrogen, USA), and reduced serum Opti-MEM® (Gibco, USA) as the growth medium. CD OptiCHO® medium (Gibco, USA), lacking HT, was used as the selection medium and was supplemented with L-Glutamine (1 mM, Sigma-Aldrich Co., USA) and selection antibiotic G418 (250 μg/mL, InvivoGen, USA). Genomic amplification of inserted vectors was carried out using MTX (Sigma-Aldrich Co., USA) in a stepwise fashion from 50 nM to 500 nM. For spinner-flask cultures, cells were sub-cultured every 48 h in T-75 flasks at 37°C and scaled up by reseeding at 0.5·10^6^ cells/ml in fresh growth medium. Both control (37°C) and hypothermic (33°C and 31°C) cultures cells were grown in chemically defined BalanCD^®^ CHO Growth A medium (Irvine Scientific, USA) supplemented with Glutamine (4 mM). Cells were cultivated in duplicate in 125 mL spinner flasks (Techne^TM^, UK) with a working volume of 60 mL, seeded at 0.8·10^6^ cells/mL and ≥ 95% viability. All cell cultures were incubated in a Forma™ Series II 3110 Water-Jacketed CO 2 Incubator (Thermo Fisher Scientific, USA) with 96% humidity and 5% CO2 enriched atmosphere. To evaluate mild hypothermic effects, cells were continuously grown at the temperature evaluated. Sampling was carried out every 24 h until viability decreased to 40%.

### Plasmid design and construction

Anti-TNFα expression vectors were constructed by cloning heavy and light variable regions from a previously obtained murine anti-TNFα antibody, to vectors encoding human immunoglobulin constant regions. The heavy chain encoding vector was constructed from pGL-4.17 (# E6721, Promega Co., USA, with neomycin resistance cassette) by incorporating a promoter and replacing the reporter gene (luc) with the gene encoding for the heavy chain. The Light chain encoding vector was constructed through a merger of pOptiVEC™-TOPO® (#12744–017, Invitrogen, USA) retaining *dhfr* with pSecTag2 B (#V900-20, Invitrogen, USA), previously modified by incorporating the light chain gene encoding the anti-TNFα antibody.

### Transfection, amplification and cloning

The parental CHO DG44 cells were grown in Opti-MEM^®^ in order to be co-transfected with the appropriate linearized plasmids by mixing the DNA stock solution (18 μg of total DNA, 9 μg of each vector) and 15 μL FreeStyle™ MAX System Reagent in a total volume equivalent to 5% of the culture volume to be transfected. After 48 h cultivation, the cells were reseeded in CD OptiCHO^®^ medium and grown until cell viability reached levels below 10%. At this point, the viable cells isolated by a Ficoll separation were seeded in 24-well plates and subsequently scaled up in T-25 flask until cell viability reached levels above 90%. For gene amplification, a methotrexate (MTX)-based method was used. The cells were seeded 0.3·10^6^ cells/ml in selection medium with MTX added in cycles. The cycles were carried out under following concentrations of MTX and G418: 1) 50 nM and 50 μg/ml, 2) 100 nM and 50 μg/ml, 3) 250 nM and 200 μg/ml and 4) 500 nM and 200 μg/ml.

To isolate clonally-derived cell lines, cells were seeded in semi-solid ClonaCell™ medium (STEMCELL Technologies Inc., Canada) according to the manufacturer’s instructions. After 15 days of cultivation, visible colonies were reseeded in 96-well plates with 100 μL of CD OptiCHO® medium. Anti-TNFα production was assessed by ELISA as described below; the top seven cell lines, in terms of anti-TNFα production, were scaled up to T-75 flasks. From those clones, two highly productive clones were selected based on maximum cell growth (CN1) and maximum anti-TNFα production (CN2).

### Analytical methods

Cell concentration and viability were determined by haemocytometer (Neubauer, Germany) using the method of trypan blue exclusion (T8154, Sigma-Aldrich Co., USA)(1:1 mixture of 0.2% trypan blue in saline and cell sample). Glucose, lactate, glutamine and glutamate concentrations were measured using a YSI 2700 Biochemistry Analyzer (Yellow Springs Inc., USA). Glutamate concentration was measured to subtract the glutamate contribution in culture medium and to reflect the real concentration of glutamine. Ammonia concentration was measured using Byosystems Analyser Y15 (BioSystems S.A., Spain) and an Ammonia AX5 kit (#12532, BioSystems S.A., Spain). Amino acids concentrations were measured by PerkinElmer Series 200 HPLC (PerkinElmer, USA) using a C-18 reversed-phase column (AccQ·Tag Column, 3.9mm×150mm, Waters, USA) and AccuTag kit (Waters, USA) according to the manufacturer’s instructions.

Anti-TNFα concentration in the medium was determined by an antigen-capture enzyme-linked immunosorbent assay (ELISA) prepared in this laboratory. ELISA 96-well plates were coated with 2.5 μg/mL of polyclonal anti chain kappa antibody (#A0191, DakoCytomation Ltd., UK) in PBS at 4°C for 16 h. The plates were then blocked in blocking buffer (PBS with 1% BSA) at 37°C for 1 h followed by additional washes with washing buffer (PBST). Samples and a infliximab (Remicade^®^, Schering Plough, USA) standard curve diluted with PBS-T/BSA were added and incubated at 4°C for 16 h. After extensive washes with PBST, Goat Anti-Human IgG H&L (HRP) antibody was added and incubated (ab97175, Abcam, USA) at 37°C for 1 h. Reactions were visualized with TMB substrate solution, and H_2_SO_4_ 1N solution addition, and the OD of each well was measured at 450 nm using a microplate reader (ELx800, BIO-TEK Instrument Inc., USA).

### Calculation of specific rates, volumetric productivity and Y_lac/glc_

The specific growth rate (*μ*) and the specific anti-TNFα productivity (q_anti-TNFα_) were determined by plotting the concentration of viable cells and anti-TNFα in medium, respectively, against integral of viable cells (IVC). The volumetric productivity (Q_anti-TNFα_) was calculated by plotting concentration of anti-TNFα in medium vs culture time. The specific glucose and glutamine consumption (q_glc_ and q_gln_) and lactate production (q_lac_) rates were determined by plotting the glucose, glutamine and lactate concentrations versus IVC throughout the culture. The corresponding *μ*, q_anti-TNFα,_ Q_anti-TNFα_, q_glc_, q_gln_ and q_lac_ were evaluated from the corresponding slopes of the plots. To evaluate the ratio of lactate produced to glucose consumed (Y_lac/glc_), lactate concentration versus consumed glucose at each time was plotted during the exponential growth phase. Y_lac/glc_ is equal to the slope of the plot.

### Gene expression analysis by RT-qPCR

RNA was extracted from cells using E.Z.N.A. ® Total RNA Kit I (Omega bio-tek Inc., USA) according to the manufacturer’s instructions. RNA concentration was quantified using Synergy 2 Spectrophotometer (BIO-TEK Instruments, Inc., USA). RNA extracts (2 μg) were treated with DNase I (Thermo Fisher Scientific, USA) to remove any trace of genomic DNA contamination following manufacturer's instructions. Reverse transcriptase production of cDNA from the RNA was performed using an Affinity Script enzyme (Agilent Genomics, USA). Quantitative PCR (qPCR) was carried out in an Agilent Mx3000P QPCR System (Agilent Technologies, USA) using a Brilliant III Ultra-Fast SYBR® Green QPCR Master Mix kit (Agilent Technologies, USA), according to the manufacturer’s instructions. All samples were run in duplicate, and no template controls were included. The thermal cycling parameters used consisted of 3 s at 95 °C as initial denaturation, followed by 40 cycles of 5 s at 95°C and 20 s at 60°C. A final extension at 72°C was carried out, followed by a melting curve to confirm primer specificity. Data were analysed using the 2-ΔΔCq method and normalized using *gapdh* as a standard. The mRNA was quantified by using the following primers (*gapdh*: forward 5’-ACGGATTTGGCCGTATTGGA-3’, reverse 5’-GCCTTGACTGTGCCTTTGAA-3’; *myc*: forward 5’- TTCGGGTAGTGGAAAACCAG -3’, reverse 5’- AGTAGAAATACGGCTGCACC -3’; *xbp1s*: forward 5’-ATGGTGGTGGTGGCAGCC-3’, reverse 5’- TCATTAATGGCTTCCAGCTTGG-3’

### Statistical analysis

Kinetics and stoichiometric parameters were calculated from at least two independent experiments and are expressed as the mean ± standard error (SE). All statistical analyses were performed with R software (version 3.1.,[43]). The significant variation of environmental and clone variables among physiological parameters and mRNA expression was evaluated by two-way ANOVA (using culture temperature– 3 levels–and clone type– 2 levels–as factors) followed by multiple comparison tests (Tukey HSD test) with normally distributed data. Significant differences in mRNA expression levels between both culture periods were evaluated by a *t*-test. Additionally, variance homogeneity and normal distribution of residuals was assessed with the Shapiro-Wilk test and visual inspection of the normal-quantile plot in order to validate the ANOVA’s assumptions. Pearson correlation was calculated between the expression levels of *xbp1s* and *anti*-*TNFα*, and q_anti-TNFα_, separately, between the expression levels of *anti*-*TNFα* and q_anti-TNFα_, as well as between the expression levels of *myc* and the maximum cell density (X_max_) in order to describe the relationship among these parameters. Principal component analysis (PCA) was performed for the main physiological parameters (i.e. 11 variables) using the ade4 package[44]. A dendrogram was built using a dissimilarity matrix (Euclidean distance) by hierarchical ascendant classification (Ward).

## Results

### Effects of mild hypothermia on cell growth and anti-TNFα production in CN1 and CN2

To investigate and compare the effect of mild hypothermia on the growth and recombinant anti-TNFα production of the two selected CHO cell lines (CN1 and CN2), cells were grown in suspension batch cultures at three different temperatures (37 (control), 33 and 31°C). The cultures were extended until cell viability decreased below 40% or the glucose in medium was completely depleted. Due to a reduction in the environmental growth temperature, changes in cell growth, viability and recombinant antibody production were observed in both cases. Two-way ANOVA indicates that cell concentration, specific growth rates, specific production rate and volumetric productivity of anti-TNFα were significantly different among culture temperatures (p < 0.05) as well as between both clones (p < 0.05) (S1 Table).

**Fig 1:**
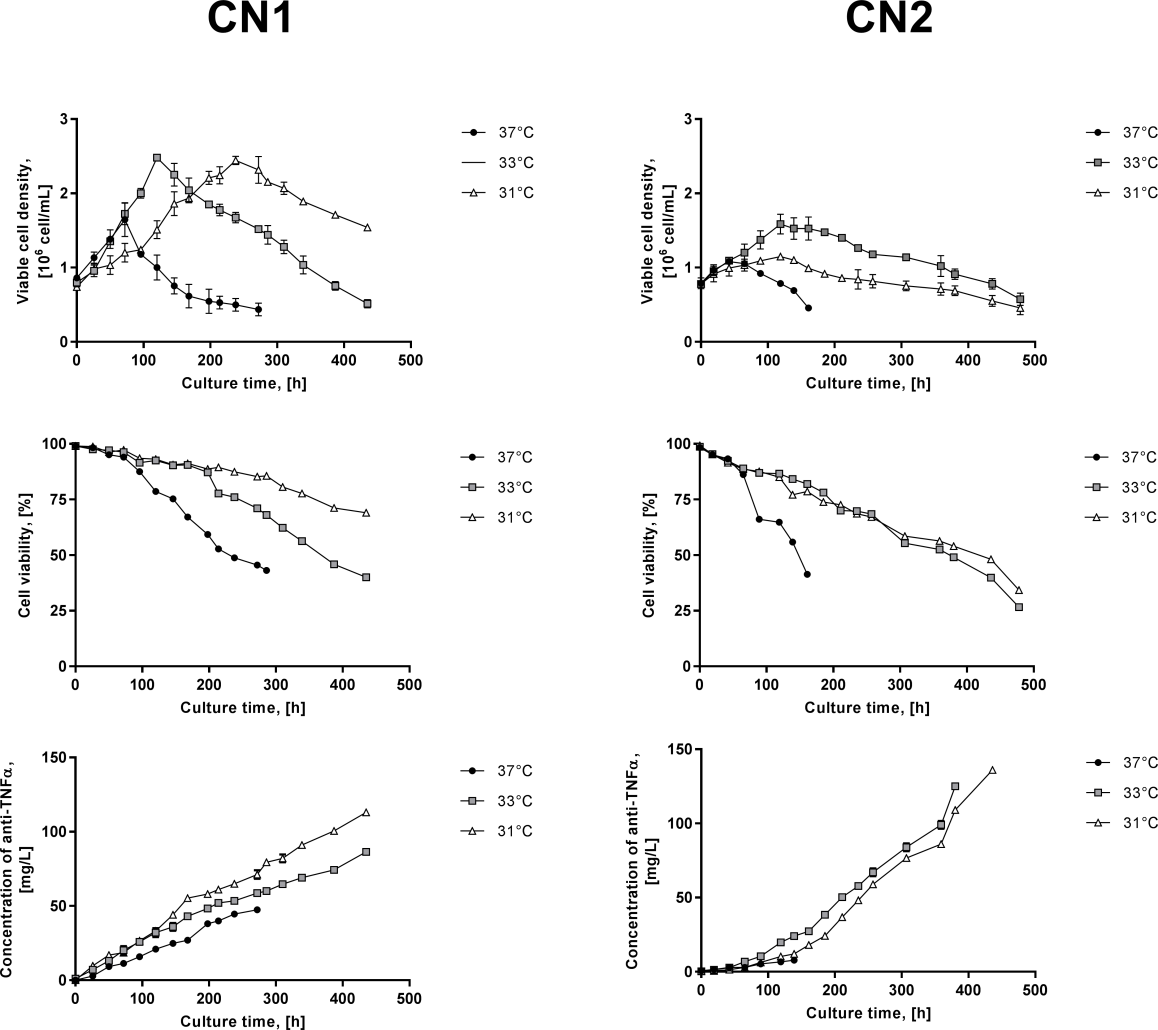
Growth, viability and anti-TNFα production profiles of CN1 and CN2 at 37°C (●), 33°C (■) and 31°C (Δ). A, B and C correspond to viable cell concentration, cell viability and concentration of anti-TNFα, respectively.

Higher cell densities were reached by cells at lower temperatures (Fig 1A). The maximum cell concentration (X_max_) of both CN1 and CN2 was found at 33°C, reaching 2.6·10^6^ cells/ml ± 0.3 in CN1 and 1.6·10^6^ cells/ml ± 0.1 in CN2. In the control cultures, in turn, the X_max_ was significantly reduced reaching 35% and 48% lower in CN1 and CN2 (S2 Table; Tukey's post hoc test, p < 0.05), respectively. Likewise cell growth, higher specific growth rates (*μ*) were significantly reached by cells at lower temperatures (Table 1; Tukey’s post hoc test, p < 0.05). The highest *μ* of both CN1 and CN2 was found at 33°C, reaching 0.204 1/d ± 0.01 in CN1 and 0.152 1/d ± 0.02 in CN2. In contrast, in cultures at 37°C, the *μ* was 11% and 44% lower in CN1 and CN2, respectively. A better culture performance (i.e. higher X_max_ and *μ*) was expected in CN1 compared to CN2 in each temperature condition considering the selection criterion of both cell lines, but the better culture performance at 33°C than 37°C was completely unexpected.

**Table 1:** Physiological and metabolic parameters of CN1 and CN2 growing at 37, 33 and 31°C.

		CN1			CN2		
Parameters	Units	37°C	33°C	31°C	37°C	33°C	31°C
μ^a^	1/h	0.0076	0.0085	0.0055	0.0035	0.0055	0.0045
X_max_^c^	10^6^ cell/mL	1.7 ± 0.2	2.5 ± 0.2	2.6 ± 0.3	0.84 ± 0.1	1.6 ± 0.1	1.1 ± 0.03
Anti-TNFα_max_^c^	mg/L	47.5 ± 2.5	86.4 ± 3.8	113 ± 9.7	15.5 ± 1.9	125 ± 0.6	136 ± 0.6
Q_anti-TNFα_^c^	mg/L/h	0.19 ± 0.01	0.20 ± 0.01	0.26 ± 0.01	0.11 ± 0.01	0.30 ± 0.02	0.35 ± 0.01
q_anti-TNFα_^c^	ng/10^6^cells/h	130 ± 4	144 ± 4	196 ± 9	172 ± 2	231 ± 25	377 ± 30
q_%VL_^c^	%viabilityloss/h	0.23 ± 0.01	0.11 ± 0.01	0.05 ± 0.00	0.32 ± 0.01	0.12 ± 0.00	0.14 ± 0. i00
Y_lac/glc_^a^	mol/mol	0.9 ± 0.2	1.0 ± 0.1	1.0 ± 0.1	2.3 ± 0.5	1.1 ± 0.1	1.3 ± 0.1
q_glc_^c^	nmol/10^6^cells/h	-105.5 ± 4.3	-70.9 ± 4.1	-64.9 ± 0.0	-135.2 ± 8.2	-66.5 ± 2.6	-70.5 ± 1.8
q_lac_^a^	nmol/10^6^cells/h	91.5 ± 4.5	83.7 ± 0.5	79.1 ± 3.0	82.3 ± 8.1	15.7 ± 1.0	25.5 ± 0.4
q_gln_^a^	nmol/10^6^cells/h	-13.7 ± 0.3	-14.8 ± 0.4	-11.8 ± 0.1	-14.4 ± 0.2	-15.3 ± 3.4	-8.7 ± 0.6
q_lac_2^b^	nmol/10^6^cells/h	-13.1 ± 0.3	-2.8 ± 0.1	-11.1 ± 0.4	n.r.	-29.5 ± 1.0	-45.8 ± 4.3
q_gln_2^b^	nmol/10^6^cells/h	n.r.	1.7 ± 0.1	2.1 ± 0.1	n.r.	7.8 ± 1.0	12.4 ± 0.8
q_NH3_^a^	nmol/10^6^cells/h	51.6 ± 3.6	60.4 ± 2.1	46.2 ± 4.3	63.2 ± 13.1	37.1 ± 1.5	31.8 ± 2.9
q_NH3_^b^	nmol/10^6^cells/h	n.r.	-3.6 ± 0.4	-2.5 ± 0.1	n.r.	-5.6 ± 1.4	-5.0 ± 0.5

^a^ Corresponding to culture period of the growing phase (S1 Fig, Supplementary information)

^b^ Corresponding to culture period of the death phase (S1 Fig, Supplementary information)

^c^ Corresponding to the complete culture time

Culture longevity was improved in cells at lower temperatures (Fig 1B). The maintenance of higher cell viability at 33°C and 31°C enabled extension of the cell cultures to at least 230 and 340 h (37% and 66%) in CN1 and CN2, respectively, as compared to 37°C. To evaluate the effect of culture temperature on cell viability, the rate of cell viability loss (q_%VL_) was calculated for each clone and temperature condition (Table 1). As expected, the maximum q_%VL_ of cells was found at 37°C, with CN1 and CN2 losing 5.5% and 7.8% viability per day in CN1 and CN2, respectively. The q_%VL_s were reduced dramatically in both cell lines as the temperature decreased showing a more acute reduction of the q_%VL_ in CN1 compared to CN2.

Total recombinant protein concentration in the medium was quantified and from these results, the specific production rate (q_anti-TNFα_) and volumetric productivity of anti-TNFα (Q_anti-TNFα_) were calculated. CN2, in particular, showed a change in the profile of anti-TNFα production after 120 h of culture, showing two different specific production rates (Fig 1). However, since the q_anti-TNFα_ and Q_anti-TNFα_ of the second stage are significantly higher than those of the first stage, only the parameters of the second stage will be considered to analyse its performance. At 37°C the q_anti-TNFα_ was 1.3- times higher in CN2 than CN1, while Q_anti-TNFα_ was 1.7- times higher in CN1 than CN2 (Table 1). These results were consistent with the clone selection criterion, since CN1 was initially selected by its growth capacity and CN2 by its production capacity. At lower temperatures, both cell lines reached significantly higher q_anti-TNFα_ and Q_anti-TNFα_ (Table 1; Tukey’s post hoc test, p < 0.05). The maximum q_anti-TNFα_ and Q_anti-TNFα_ of both cell lines was at 31°C, in CN1 they were 195 ng/10^6^cell/h ± 9.5 and 0.26 mg/L/h ± 0.001, whereas in CN2 377 ng/10^6^cell/h ± 30.5 and 0.35 mg/L/h ± 0.008. These results correspond to an increase of 28% in q_anti-TNFα_ and of 27% in Q_anti-TNFα_ in CN1 compared to the control, while the increases of these parameters in CN2 were sharply pronounced, with q_anti-TNFα_ increasing 81% and Q_anti-TNFα_ 85% compared to 37°C.

### Effects of mild hypothermia on cellular metabolism in CN1 and CN2

**Fig 2:**
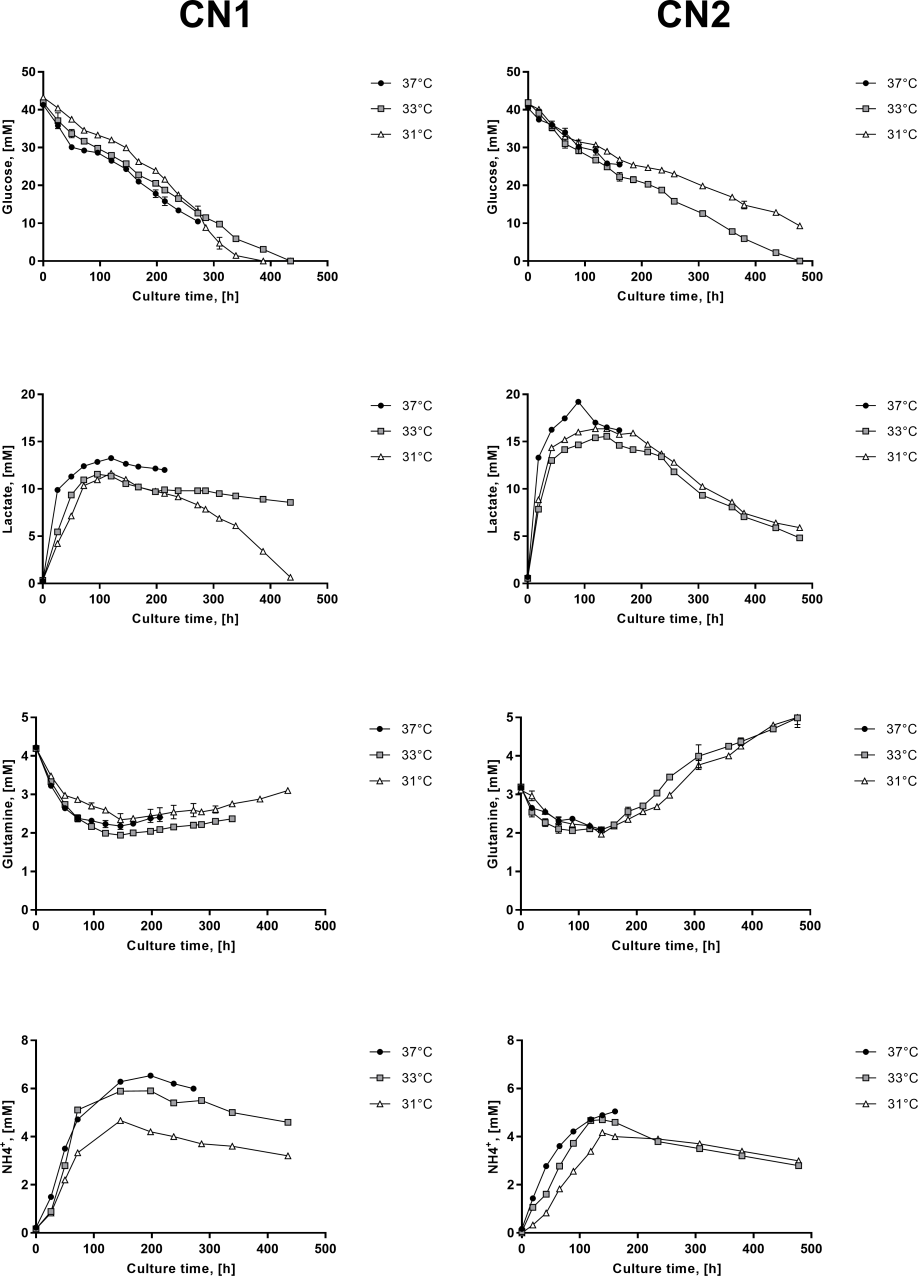
Glucose, lactate, glutamine and ammonia profiles of CN1 and CN2 growing at 37°C (●), 33°C (■) and 31°C (Δ). A, B, C and D correspond to concentrations in medium of glucose, lactate, glutamine and ammonia, respectively, in mM.

To evaluate the effects caused by temperature reduction on cellular central metabolism (CCM) glucose, lactate and glutamine were monitored throughout cultures. Additionally, concentration of 11 amino acids in medium were measured at 0 (fresh medium), 72 and 120 h in order to obtain a more detailed perspective of the metabolic profile of CN1 and CN2 at each condition (S2 Fig). By evaluating the corresponding specific rates of consumption and production, it was noted that variations in culture temperature and clone type significantly affected these parameters (S1 Table, two-way ANOVA p < 0.05), and thus cell metabolism (Fig 2).

Glucose utilization was strongly decreased by a reduction of temperature (Fig 2A). As expected, both cell lines reached its maximum specific consumption rate of glucose (q_glc_) at 37°C (105.5 nmol/10^6^cells/h ± 4.4 in CN1 and 135.2 nmol/10^6^cells/h ± 8.2 in CN2). As the culture temperature decreased, cells reduced the glucose consumption. The lowest q_glc_s were registered at 31°C, representing a decrease of more than 60% and 50% in CN1 and CN2, respectively, compared to the control (Table 1). In addition to the temperature effect, it is interesting to note that specific consumption rates of glucose remained constant during the culture time for all condition despite the subsequent change in cell metabolism.

Interestingly, lactate and glutamine profiles showed a simultaneous metabolic shift, from production to consumption in lactate and from consumption to production in glutamine, after 120 h of culture in all evaluated conditions (Fig 2B and 2C). Given this dual behaviour, they were analysed in two stages (before and after shift). In the first stage, compared to 37°C, the specific production rate of lactate (q_lac_) in CN1 was slightly reduced reaching a reduction of 9% and 13% at 33°C and 31°C, respectively, whereas in CN2 the reduction of q_lac_ was more acute reaching 81% and 69% lower at 33°C and 31°C, respectively. For glutamine, in turn, CN1 showed no significant difference in its specific consumption rate of glutamine (q_gln_) among temperatures, while CN2 showed slight changes in q_gln_ at lower temperatures, increasing at 33°C and decreasing at 31°C compared to 37°C. In the second stage, both CN1 and CN2 started consuming lactate and producing glutamine (Fig 2). CN1 showed considerably low specific lactate consumption rates (q_lac_2) consuming approximately 10% of the lactate produced in all culture temperature conditions, whereas CN2 showed higher q_lac_2 values that were exacerbated by lower temperature. In contrast, CN1 showed slightly increased specific production rates of glutamine (q_gln_2) as the temperature decreased, while in CN2 the increase of q_gln_2 was surprisingly high producing 67% more glutamine than the initial amount at 33°C and 31°C. Clearly, CN2 was the cell line experiencing the most marked effect on its metabolic parameters as a result of the culture temperature reduction before and after the shift.

Since the ratio of lactate produced to glucose consumed (Y_lac/glc_) gives us relevant indications of the cellular metabolic state, it was calculated for all conditions before the shift (Table 1). As a result of culture temperature reduction, CN1 showed no significant differences in Y_lac/glc_, while it considerably declined in CN2, reaching a reduction of 67% and 63% at 33°C and 31°C, respectively, compared to the control.

Ammonia was produced in larger amounts in CN1 than CN2 both at 37°C and low temperatures (Table 1). Low temperature in cultures reduced the production of ammonia in both clones, except for CN1 at 33°C. As with glutamine profile, ammonia also presented a shift of production to reuse after 120 h of culture in both cell lines (Fig 1D). The reuse of ammonia was observed in both cell lines, only in cultures at low temperature, and was greater in CN2 than CN1. These results coincided with the simultaneous metabolic shift of glutamate and were consistent with the increased generation of glutamine production. Amino acids profile indicated that most amino acids are consumed in all conditions, except for alanine in CN1 and CN2 and glutamate exclusively in CN2 at the time of metabolic shift (S2 Fig). Alanine production over culture time is in agreement with previous reports [45], while glutamate production in CN2 was rather unusual and probably explained by the increase of glutamine at this time.

### Differential expression of mRNAs encoding for Anti-TNFα, Myc and XBP1s in CN1 and CN2

**Fig 3:**
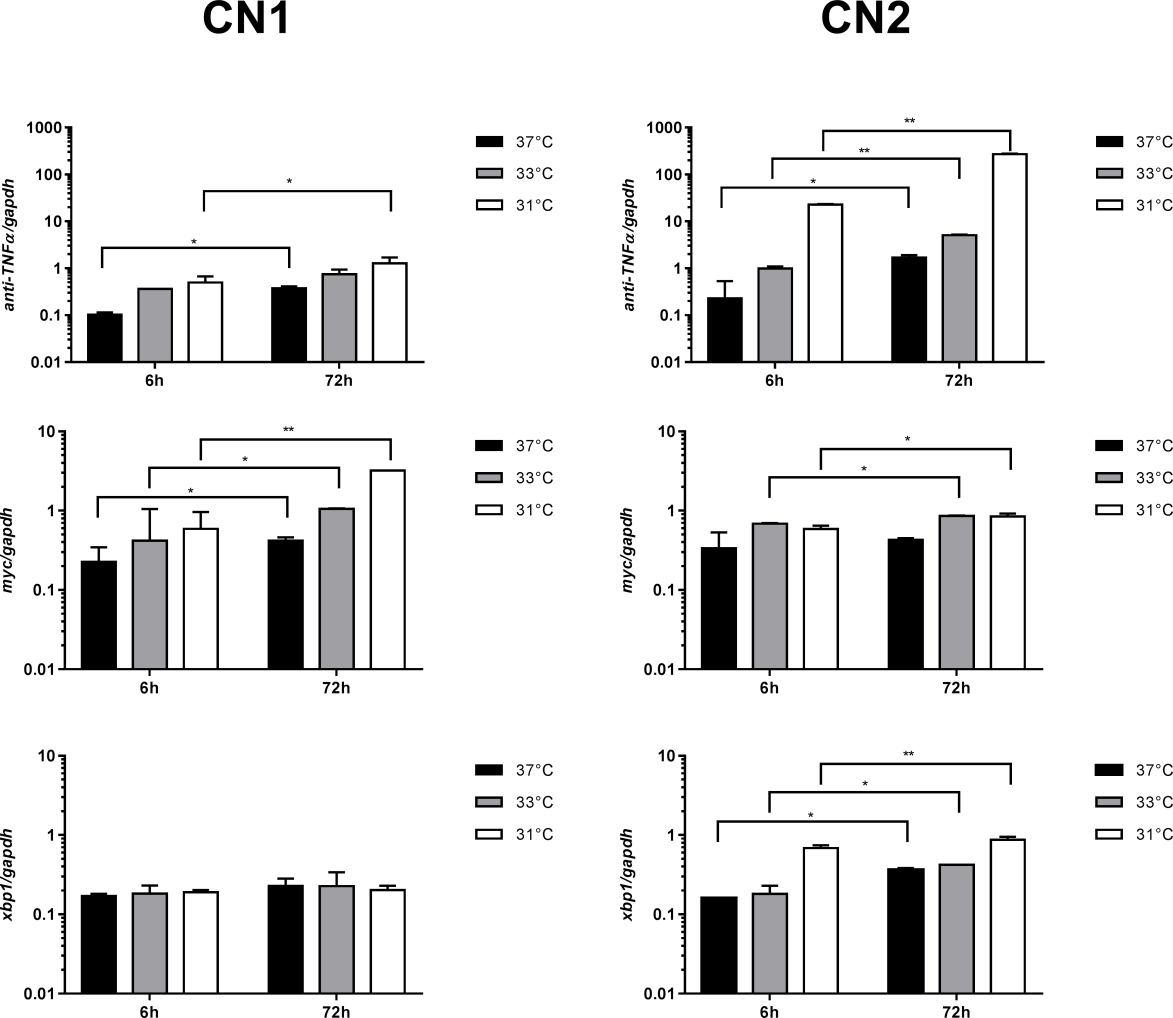
Differential expressions of mRNA encoding for anti-TNFα, Myc and XBP1s in CN1 and CN2 at 37°C (■), 33°C (■) and 31°C (■). The relative expressions of *anti-TNFα*, *myc* and *xbp1s* were analysed at 6 and 72 h of culture using RT-qPCR and *gapdh* as reference gene. The results are expressed and plotted in log_10_. Significant differences (α = 0.05) between temperature and clones according to the Tukey HSD test are indicated by the p-values in the S5 Table (Supplementary file). Differences in the expression levels of *anti-TNFα*, *xbp1s* and *myc* between both culture periods (i.e. 6 h vs 72 h of culture) were evaluated by a *t*-test (S4 Table, Supplementary file) and expressed in the figure as: * p < 0.05, ** p < 0.01 and p < 0.001.

To understand the effects of culture temperature reduction on anti-TNFα production and cellular metabolism of CN1 and CN2 at the transcriptional level, the differential expression of mRNAs encoding for anti-TNFα, Myc and XBP1s was analysed in each condition at 6 and 72 h of culture. To do so, the ratio of the relative mRNA content of each gene with respect to the *gapdh* was calculated (Fig 3). Variance analysis (two-way ANOVA) reveals that the relative expression of mRNA encoding for anti-TNFα, Myc and XBP1s were significantly different among culture temperatures (p < 0.05) as well as between both clones (p < 0.05) (S3 Table). Additionally, differences in the expression levels of *anti-TNFα*, *xbp1s and myc* between both culture periods (i.e. 6 h vs 72 h of culture) were evaluated by a *t*-test and showed statistically significant variations (S4 Table). The differences in expression levels between both culture periods were mainly associated with cellular acclimation processes of CN1 and CN2 to lower culture temperatures after inoculation.

Differences between CN1 and CN2 in the levels of mRNA expression were highlighted by Tukey's post hoc test, showing a significantly higher expression of *anti-TNFα* (p < 0.05) (p < 0.05) in CN2 compared to CN1 at both culture periods. As observed in Table 1 and Fig 3, higher expression levels of *anti-TNFα* coincide with a greater production of Anti-TNFα. The expression levels of *xbp1s* at 6h of culture only presented significant changes between CN1 and CN2 at 31°C (p < 0.05), while at 72h of culture CN2 showed a significantly higher expression of *xbp1s* at all culture temperature. The expression levels of *myc* at 6 h of culture showed no major differences between both cell lines (S5 Table; Tukey's post hoc test, p > 0.05). However, the results indicated that differences between CN1 and CN2 in *myc* expression were enlarged at 72 h, showing a significant increase in the expression level of *myc* in CN1 compared to CN2 at both 33°C and 31°C (S5 Table; Tukey's post hoc test, p < 0.05). These differences in *myc* expression were observed only under mild hypothermic growth, while at the control condition CN2 and CN1 did no present significant variations.

When assessing the temperature effect regardless of the cell line, the results revealed that the temperature reduction led to a significant increase in the expression level of *anti-TNFα* and *myc* in CN1 and CN2 (S5 Table; Tukey’s post hoc test, p < 0.05), whether it was reduced from 37°C to 33°C or from 33°C to 31°C, at both culture periods. The expression level of *xbp1s* did not show significant changes with the temperature reduction in CN1, while in CN2 it was significantly higher at 31°C where the highest *anti-TNFα* expression (Fig 3) and anti-TNFα production (Table 1) were reached. These results were coherent with those reported in the literature and with the metabolic state of cells during mild hypothermia. In fact, since *xbp1s* is involved in the regulation of synthesis and secretion of proteins and those processes are enhanced when cells are exposed to lower physiological temperatures [27,28], higher expression of *xbp1s* is predicted at lower culture temperature and at more advanced period of cultures when cells are totally acclimated to this conditions. The same occurred with *anti-TNFα*, which was expected a higher expression during hypothermic growth.

Pearson’s correlation showed that the expression level of *xbp1s* has a positive relationship with the expression level of *anti-TNFα* (R_6h_ = 0.99, p < 0.05; and R_72h_ = 0.94, p < 0.05) and q_anti*-TNFα*_ (R_6h_ = 0.9, p < 0.05; and R_72h_ = 0.86, p < 0.05), respectively. Additionally, the expression level of *anti-TNFα* was positively correlated to q_anti*-TNFα*_ (R_6h_ = 0.86, p < 0.05; and R_72h_ = 0.86, p < 0.05), while the expression level of *myc* was positively correlated to X_max_ (R_72h_ = 0.82, p < 0.05) only at 72 h. Therefore, the upregulated expression of *xbp1s* and *myc* as well as overexpression of *anti-TNFα* encoding mRNAs coincides with the results of culture performance (Table 1; Fig 3). Namely, cells presented the highest expression of *xbp1s* and *anti-TNFα* at the most productive condition, while at the better cell growth condition cells presented the higher expression levels of *myc*.

### Multivariate analysis of physiological parameters in CN1 and CN2 under mild hypothermic conditions

A principal component analysis (PCA) of the physiological parameters of CN1 and CN2 was performed to elucidate the influence of these variables on the cell behaviour. Eleven variables including X_max_, *μ*, Y_lac/glc_, q_anti-TNFα_, anti-TNFα_max_, Q_anti-TNFα_, q_glc_, q_lac_, q_gln_, q_lac_2 and q_gln_2 were considered for this analysis. To those parameters which there were no data, namely qlac2 in CN2 and qgln2 in both clones at 37°C, PCA was performed replacing these parameters by zero. This assumption delivered a more robust and consistent result than without considering these both parameters, representing better the variability of the system. The parameters were obtained and calculated from CN1 and CN2 cells cultured at 37, 33 and 31°C.

**Fig 4:**
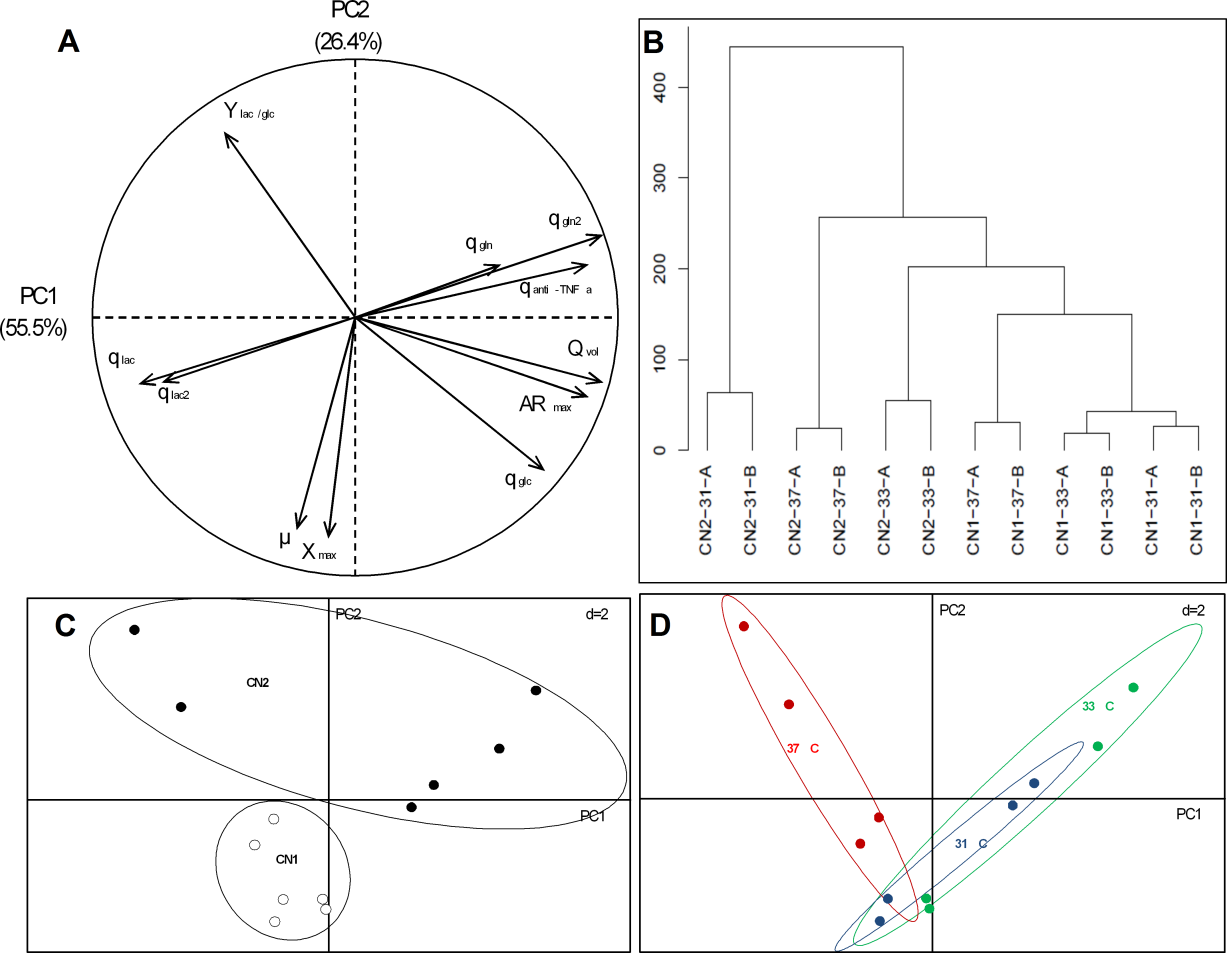
Principal component analysis (PCA) using 11 physiological parameters of CN1 and CN2 at 37, 33 and 31°C. The variables analysed were maximum cell density (X_max_), specific cell growth rate (*μ*), yield of lactate from glucose (Y_lac/glc_), specific anti-TNFα productivity (q_anti-TNFα_), maximum anti-TNFα production (anti-TNFα_max_), volumetric productivity (Q_vol_), specific consumption rate of glucose (q_glc_), glutamine (q_gln_), and lactate after shift (q_lac_2); specific production rate of lactate (q_lac_) and glutamine after shift (q_gln_2). A: correlation circle showing relation between the measured parameters; B: Cluster analysis (Ward’s method) of the observations using Euclidian distance; C and D: Ordination of the first and second principal component (PC1 and PC2, respectively), showing observation distribution. Different conditions are plotted using circles to identify the effect of clones (C) and temperature (D).

**Fig 5:**
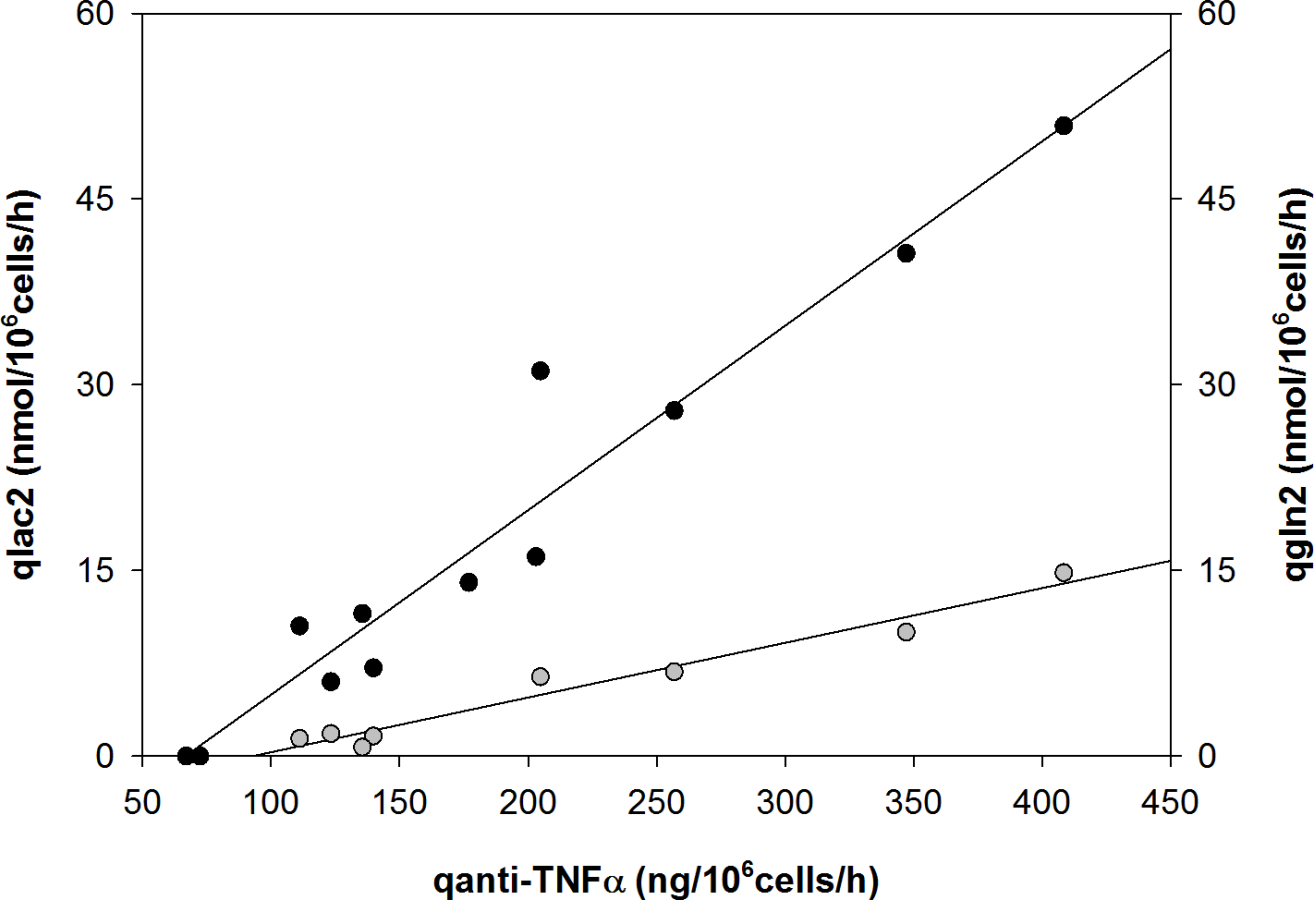
Relationship between q_anti-TNFα_ and q (lactate: ●, and glutamine: ●; both after shift).

In the PCA, principal components 1 and 2 (PC1 and PC2, respectively) represented 82% of the total variance (Fig 4). By evaluating the influence of each observation loaded in the PCA matrix, the parameters q_gln_2, q_lac_2 and q_anti-TNFα_ represented those that had a greater influence on the total variance. To understand the relationship among these parameters, the corresponding data were plotted in a two-dimensional diagram considering PC1 and PC2 (Fig 4A). From this, a positive correlation between q_gln_2 and q_anti-TNFα_ was determined, as was an indirect correlation between q_gln_2 and q_lac_2 and between q_anti-TNFα_ and q_lac_2 (Fig 5). It has to be emphasized that these relationships are more meaningful for mild hypothermia due to the assumptions of q_gln_2 and q_lac_2 at 37°C.

In addition, the ordinations B and C showed clear differences between the control and hypothermic conditions as well as between sample from CN1 and CN2. This result was confirmed using clustering analysis (Fig 4B), which indicated the presence of these two separate clusters in each case (Fig 4C and 4D).

## Discussion

One of the main environmental variables to manipulate in cell cultures is the temperature, which affects both growth and protein production of CHO cell lines. In the literature, it is widely reported that recombinant CHO cells grown in batch culture at low temperatures show a decrease in growth and an increase in protein production [14,21,22,29,46]. However, the impact of changes in the expression of transcriptional regulatory factors, such as *myc* and *xbp1s*, due to hypothermic conditions on cell growth and protein production has not been studied yet. For this purpose, we selected two anti-TNFα producing cell lines as models with two different phenotypic features (i.e. CN1 with high proliferation and CN2 with high specific anti-TNFα productivity) and investigated how changes in their cell growth, anti-TNFα production and carbon metabolism by reduced culture temperature may be associated with changes in the expression levels of *myc* and *xbp1s*.

During hypothermic growth (33°C and 31°C), cell growth was improved in CN1 and CN2, but to a different degree. The same occurred with the *μ* which reached its maximum level at 33°C instead of 37°C in both cell lines, but with notably low values in all cases (i.e. less than 0.01 1/h). The higher *μ* and maximum viable cell density reached at low temperature in CN1 and CN2 certainly contrasts with the literature where a reduction of these both parameters is usually observed. In previous cases, an slight improvement of cell growth using sub-physiological temperatures (< 37°C), usually ranging from 36.5°C to 35°C, has been observed in mammalian cell culture [47–49]. Along with this, Yoon et al.[18] and Sunley et al.[19] showed an improved cell growth and increased *μ* in recombinant CHO cells adapted to low temperatures compared to non-adapted cells (temperature shift). So, one possible explanation for the improved cell growth of CN1 and CN2 might be the expression of certain cold–inducible gene products that would act as an aid for cells to avoid the early cell growth decline. Low culture temperatures also improve viability, which reduced the rate of viability loss (Table 1) and prolonged the growth phase of CN1 and CN2 in at least 50% more than the control cultures (Fig 1). The improved viability and extension of the growth phase during mild hypothermia were consistent with the previous reports in CHO cells [18,21,50], and would probably explain the higher maximal viable cell densities reached for these clones at low temperatures by positively impacting their ICVs. These results suggested a new benefit of using mild hypothermia that may help culture highly productive cell lines with a high rate of viability loss. The extent of growth improvement in both cell lines at low temperature compared to 37°C is certainly a novel fact, not previously reported in CHO cells, that should be considered for future research.

The reduction of culture temperature down to 31°C promoted anti-TNFα production and enhanced the q_anti-TNFα_ in both CN1 and especially CN2 (Table 1). The increase of the production of anti-TNFα and q_anti-TNFα_ in both cell lines is consistent with findings previously reported in the literature [14–17,51], where at lower temperatures, the production and specific recombinant protein productivity were considerably increased in CHO cell batch cultures. Mild hypothermia consistently increased the differential expression of *anti-TNFα* in CN1 and CN2, and extended the differences in the *anti-TNFα* levels between both clones observed at 37°C (Fig 3). An increase in the mRNA abundance of recombinant genes due to low temperatures has been previously reported in different CHO cell lines and linked to an increase in productivity of recombinant proteins [16,21,52–54]. The overexpression of the recombinant gene at low temperatures however seems to depend on the specific cell line and recombinant protein, because other studies have shown no increase in the recombinant gene expression [29,55]. While the exact reason of why expression levels of recombinant genes increase under mild hypothermia still remains unknown, recent studies indicated that under these conditions there is an increase in the mRNA stability [10,16,21,56], which would maintain greater mRNA abundance of recombinant genes for longer periods and thus lead to a larger protein synthesis. In addition, the results revealed a direct correlation between expression levels of mRNA encoding anti-TNFα and the q_anti-TNFα_ (Pearson’s correlation, p < 0.05 at 6 and 72 h), clearly indicating that high anti-TNFα productivity was strongly related to the overexpression of *anti-TNFα*. This also suggested that the higher production anti-TNFα in CN2 compared to CN1 was due to the difference in expression of the recombinant *anti-TNFα*. While the explanation of these phenomenon takes places in a multivariable context including gene expression and the corresponding response of the cell metabolism to those changes [10], this study corroborates that hypothermic growth provides appropriate conditions to enhance recombinant protein production, particularly anti-TNFα, in which the overexpression of recombinant genes play a central role.

Both cell lines cultured at lower temperatures upregulated the expression of *myc* and its higher expressions occurred at culture condition reaching higher cell densities. The upregulation of *myc* is consistent with the previously reported in a t-PA producing CHO cell lines under mild hypothermia conditions, where a 3.4-fold increase 48 h after temperature down shift was observed [29]. Myc regulates key elements involved in cell proliferation and carbon metabolism [30], and its upregulation might apparently stimulate cell growth, glycolysis, glutaminolysis and lactate production. The current data indicated that in conditions presenting a *myc* upregulation, CN1 and CN2 reached the higher cell densities (Table 1) and presented a direct correlation between *myc* vs X_max_ (Pearson’s correlation, p < 0.05). However, it was not possible to observe a further improvement in the metabolic efficiency in any condition where *myc* was upregulated. While the observed correlation between *myc* vs X_max_ is certainly interesting and in compliance with the observed by Ifandi & Al-Rubeai [31], where higher maximal viable densities were achieved in CHO cell lines overexpressing *myc*, it does not mean that just endogenous *myc* upregulation improves the growth capacity in cells. As previously discussed, the differences in the maximal viable densities among cultures were probably related to the levels of rates of viability loss showing CN1 and CN2 in cultures (Table 1). The results showed a higher expression level of *myc* in CN1 than CN2 for all conditions (Tukey post-hoc test; p < 0.05), and this coincided with the higher maximal viable densities in CN1 than CN2. In another study, Ifandi & Al-Rubeai [57] showed that a cell line transfected with *myc* exhibited apoptosis at much lower rates than the parental CHO cell line, despite the widely reported proapoptotic features that confer the overexpression of *myc*. The notable reduction of rate of viability loss in CN1 compared to CN2, and at 33°C and 31°C compared to 37°C (Table 1), suggests that the upregulation of *myc* at low temperatures might improve cell viability and thus make the difference between the maximal viable cell densities reached in CN1 and CN2. This idea was supported by the inverse relationship between the expression levels of myc and q%VL (Pearson’s correlation, R_72h_ = -0.75, p < 0.05). Nevertheless, we cannot rule out the possibility that changes in the expression of other not evaluated transcriptional factors or genes due to the low temperature have affected cell growth or acted simultaneously with *myc*.

While no changes in the expression of *xbp1s* were observed in CN1, CN2 showed a large upregulation of *xbp1s* under mild hypothermia, particularly at 31°C. Currently, changes in *xbp1s* expression during hypothermic growth has been not assessed, but Bedoya-López et al. [29] observed a large upregulation of genes coding for proteins involved in protein synthesis and processing of the ER and Golgi under mild hypothermia. We also observed direct correlations between the *xbp1s* expression levels and the expression of *anti*-*TNFα* (Pearson’s correlation, p < 0.05 at 6 and 72 h) and specific anti-TNFα productivity (Pearson’s correlation, p < 0.05 at 6 and 72 h) that might suggest that the *xbp1s* upregulation was promoted by the production of anti-TNFα. While these correlations were correctly matched to the results of CN2, which showed at 33°C and 31°C a large upregulation of *xbp1s* and a 1.34 and 2.2-fold increase in the q_anti-TNFα_, the expression of *xbp1* in CN1 did not vary significantly despite the 1.5-fold increase in the q_anti-TNFα_ at 31°C. The differences in the *xbp1s* expression between both clones seem to be associated with the specific anti-TNFα production levels during mild hypothermia, where the q_anti-TNFα_ at 31°C of CN1 and CN2 were 196 and and 377 ng/10^6^cells/h, respectively. This suggests that there might be a “load threshold” on the ER triggering the upregulation of *xbp1s*. XBP1s is key in the expression of proteins related to secretory pathways [33] and its upregulation may boost the secretory machinery of cells [37,58,59]. In this context, several studies have attempted to overexpress *xbp1s* in CHO cells, but with mixed results [36–38,40–42,59–61]. Ku et al. [59] reported no effects of *xbp1s* overexpression on EPO productivity in stable cell lines but significantly enhanced transient production in EPO-saturated CHO cells, while Pybus et al. [61] showed that the effects of *xbp1s* overexpression were more pronounced on CHO cells expressing ‘difficult-to-express’ r-proteins with limiting folding and assembly reactions, than cells expressing an ‘easy-to-express’ r-protein. Therefore, the upregulation of *xbp1s* in CN2 at low temperature, particularly at 31°C, seems to be a cellular response to a feasible overload of the ER and secretory machinery of cells, caused by the increase of protein production. However, a thorough study on this topic should be done to provide enough data to support this idea.

Another relevant point with respect to production of recombinant protein is the dependence of specific protein productivity from certain physiological parameters. Several studies have reported improvements in production of recombinant protein in mammalian cells batch cultures under conditions of hypothermic growth and low specific growth rate[18,19,62], indicating a correlation between increasing specific productivity of a recombinant protein and decreasing the specific growth rate. However, the present results showed no apparent relation between those parameters (Fig 4). In fact, the production of anti-TNFα was directly and indirectly correlated to q_gln_2 and q_lac_2 (Fig 5), respectively, suggesting that variations in q_anti-TNFα_ under mild hypothermia were more closely associated with changes in carbon metabolism instead of specific growth rate.

Cell metabolism was globally affected by reducing the culture temperature, noticing a turning point in the lactate and glutamine metabolism. In the first stage, the decreased q_glc_, q_lac_ and q_gln_ values in both cell lines at lower temperatures were expected since similar reductions in cellular metabolism have been previously reported for recombinant CHO cells cultured under conditions of mild hypothermia [53,63]. Moving forward in culture time, CN1 and CN2 exhibited a simultaneous metabolic shift of lactate and glutamine in all conditions. The consumption of lactate has been widely reported in CHO cell culture both in presence of glucose and under conditions of very low or null glucose concentration [45,64–70]. When this occurs, a decline of cell growth, a notable reduction of the specific consumption rates of glucose and amino acids, and a great improvement in product titre have been observed [64,69–71]. Our results were consistent with the timing of cell growth decline, reduction of glutamine consumption and the increase of anti-TNFα production, but they differed from the reduction in glucose consumption which remained constant throughout the all cultures. Moreover, the simultaneous metabolic shift between lactate and glutamine has been less common, only reported in two different CHO cell lines and both under mild hypothermia [53,65]. To elucidate the causes triggering this phenomenon, understanding the redox metabolism and the relationship between lactate and glutamine becomes paramount [65]. Several studies have suggested that the metabolic shift of lactate reflects a disruption in the balance between cytosolic supply and mitochondrial demand for reducing equivalents [65,67,70]. In mammalian cells, lactate production is mainly related to glucose uptake and NADH levels, with changes in lactate levels noted as these parameters vary [72]. Nolan and Lee [65] stated that a shortage in the NADH supply reverses the net flux of redox reactions such as lactate dehydrogenase, which would add to the supply of reducing equivalents in the cytosol, and rebalance the cytosolic and mitochondrial redox fluxes. Furthermore, Zagari et al. [68] suggested that glutamine and glutaminolytic pathway play an important role in modulating the lactate production and the flux through the tricarboxylic acid (TCA) cycle. These observations are consistent with the results of the PCA showing a direct relationship between q_lac_ and q_gln_ (before and after the metabolic shift) (Fig 4A). A recent flux balance analysis of CHO cells showing the distribution of the intracellular fluxes before and after a metabolic switch from lactate production to consumption indicated a large reduction of the flux through the TCA cycle and ATP production in cells under lactate consuming metabolic state [70]. Although we cannot elucidate the causes of the occurrence of this metabolic shift with our current experimental data, a plausible unbalance in the ratio NAD^+^/NADH, particularly a reduction in the generation of NADH due to the low flux through TCA cycle and glutamine production, might explain the simultaneous metabolic shift in lactate and glutamine. The metabolic shift of cells from a producing to a consuming lactate state deserves an in-depth analysis, since both the presence and the magnitude of this shift might be relevant metabolic indicators for highly productive CHO cell lines.

The current results also show an increase of anti-TNFα production along with this metabolic shift, particularly in CN2. As cells stopped using glutamine as a secondary carbon and energy source and replaced it with lactate, the anti-TNFα production was also enhanced in CN1 and particularly CN2. The relationship between q_anti-TNFα_ and either q_lac_2 or q_gln_2 is described in Fig 5 where an increased q_anti-TNFα_ was directly and indirectly correlated to an increased q_lac_2 and q_gln_2, respectively. These results are in compliance with those studies describing an increase in recombinant protein production after the metabolic shift of lactate from production to consumption [64,69–71]. Certain metabolic elements related to central carbon metabolism, particularly the balance between glycolysis and TCA, are crucial indicators to the both growth and productivity[73], providing the potential to direct cell function towards a more productive process via the development of better cell lines [74]. In this context, it is worth pointing out the strong relationship between anti-TNFα production and specific lactate consumption, since previous studies suggested that lactate utilization in recombinant CHO cell cultures may offer an indicator of a productive process [69]. While there is a strong relationship between those parameters, the emergence of the simultaneous shift is likely a consequence of global changes in cell metabolism under hypothermic conditions that might be mediated through changes in the expression levels of hundreds of genes, including the transcriptional regulatory factors *myc* and *xbp1s*. To support this idea, however, a more detailed research on the transcriptome of CN1 and CN2 under mild hypothermia is required.

## Conclusion

In this study, we could underline the intrinsic differences between two anti-TNFα producing cell lines (CN1 and CN2), both in their capacity of express a recombinant gene and all their physiologic signatures (i.e. cell growth, viability, nutrient consumption and waste production), that make the process of clone selection a very important aspect in the development of cell lines. The impact of mild hypothermia on cell growth, metabolism, recombinant protein production and expression of *myc* and *xbp1s* were successfully assessed in both anti-TNFα producing CHO cell lines. Low temperature improved cell viability prolonging the growth phase in all cases, and increased de expression of the recombinant *anti-TNFα* and the production of anti-TNFα in both clones. CN2 showed a better production of anti-TNFα than CN1 in all cultures and these differences were mainly associated with the capacity of expressing the recombinant gene. Direct correlations between the expression of *myc* and the maximal viable cell density, and between the expression of *xbp1s* and the specific anti-TNFα productivity were observed in both clones under mild hypothermia. The upregulation of *myc* under mild hypothermia coincided with improved cell growth and viability of CN1 and CN2. Meanwhile, the increased expression of *xbp1s* at low temperature may respond to the large anti-TNFα production, particularly in CN2 at 31°C. The current study presents novel findings regarding the regulation of the *myc* and *xbp1s* under mild hypothermia and raises the question whether the simultaneous ectopic expression of these transcriptional factors may be interesting target for CHO cell engineering and result in improvements of culture performance. Moreover, a simultaneous metabolic switch of lactate and glutamine was observed in CN1 and CN2, and its extension coincide with an increase in the anti-TNFα production, particularly CN2. A deeper understanding of the molecular difference of these clones might be an interesting issue for further studies that should consider their transcriptome characterization and the identification of key metabolic indicators of the culture performance.

## Supporting information

S1 Fig Viable cell densities (XV, 106 cell/mL) vs integral of viable cell (IVC) of CN1 and CN2 at 37, 33 and 31°C. *Light grey* areas represent the growing phase of each culture. *Dark grey* areas represent the death phase of each culture. The period of time used for the calculation of specific rates (Table 1) are detailed on the top of each area.
(TIF)

S2 Fig Amino acid concentration (mM) in medium of CN1 (left) and CN2 (right) at 37, 33 and 31°C. Bars at each temperature correspond to the concentration of samples taken at 0, 72 and 120h.
(TIF)

S1 Table Impact of clone type and temperature on physiological parameters (two-way ANOVA factors; n = 3). (DOCX)

S2 Table Tukey HSD test for the comparison of physiological parameters between clone type and culture temperature samples. (DOCX)

S3 Table Impact of clone type and culture temperature on the differential expressions of mRNA encoding for anti-TNFα, Myc and XBP1S at 6 and 72h (two-way ANOVA factors; n = 3). (DOCX)

S4 Table T-test of the differential expressions of mRNA encoding for anti-TNFα, Myc and XBP1S between 6 and 72h in CN1 and CN2 at 37, 33 and 31°C. (DOCX)

S5 Table Tukey HSD test for the comparison of the differential expressions of mRNA encoding for anti-TNFα, Myc and XBP1S at 6 and 72h between clone type and culture temperature samples. (DOCX)
