# Supplementary figures and images for: Mild hypothermia upregulates myc and xbp1s expression and improves anti-TNFα production in CHO cells
Source: PLoS One. 2018 Mar 22;13(3):e0194510. doi: 10.1371/journal.pone.0194510 (PMC5864046; doi:10.1371/journal.pone.0194510)

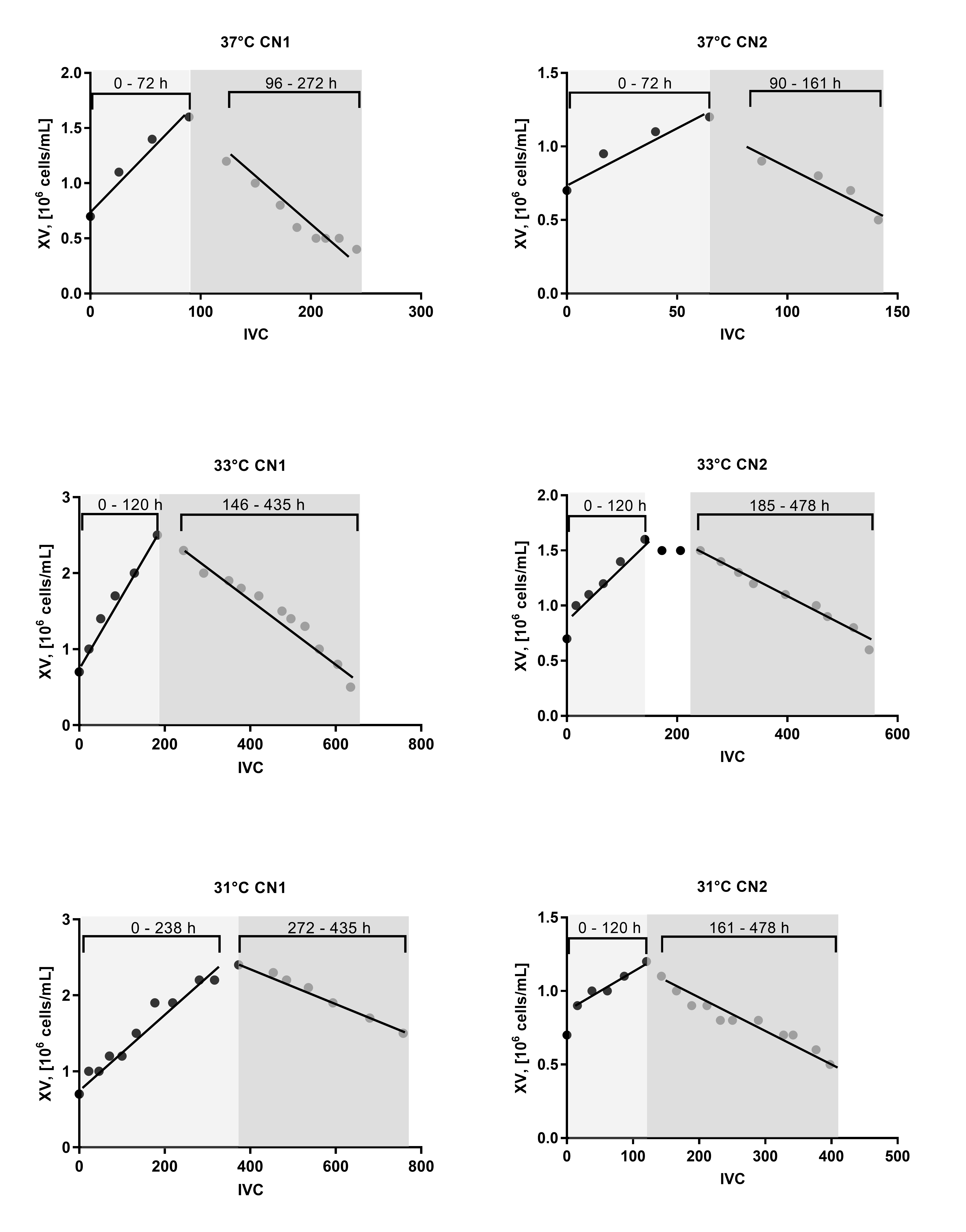

Supplement: S1 Fig — Light grey areas represent the growing phase of each culture. Dark grey areas represent the death phase of each culture. The period of time used for the calculation of specific rates (Table 1) are detailed on the top of each area. (TIF) [file pone.0194510.s001.tif]

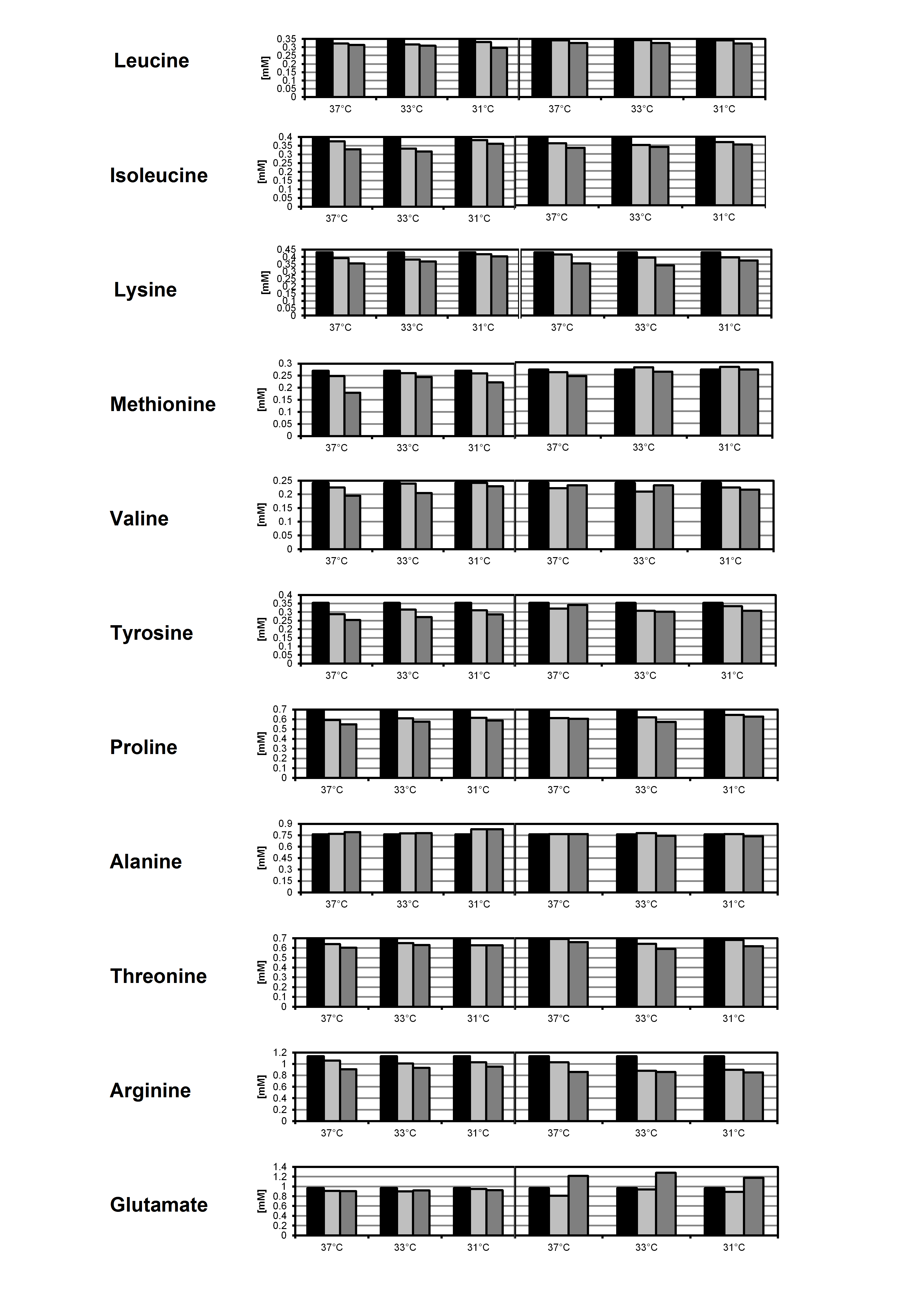

Supplement: S2 Fig — Bars at each temperature correspond to the concentration of samples taken at 0, 72 and 120h. (TIF) [file pone.0194510.s002.TIF]
