# Supplementary material for: Mild hypothermia upregulates myc and xbp1s expression and improves anti-TNFα production in CHO cells
Source: PLoS One. 2018 Mar 22;13(3):e0194510. doi: 10.1371/journal.pone.0194510 (PMC5864046; doi:10.1371/journal.pone.0194510)
Supplement: S1 Table — (DOCX) [file pone.0194510.s003.docx]

S1 Table. Impact of clone type and Temperature on physiological parameters (Two-way ANOVA factors; n=3).

| **Parameters** | **Clon** | | **Temperature** | | **Clon:Temperature** | |
| --- | --- | --- | --- | --- | --- | --- |
|  | **F value** | **P value** | **F value** | **P value** | **F value** | **P value** |
| U | 15.289 | 0.00789 | 4.339 | 0.0683 | 1.475 | 0.30128 |
| Xmax | 53.444 | 0.000334 | 8.21E+00 | 0.019193 | 2.64E+00 | 0.150401 |
| Ylac/glc | 6.981 | 0.0384 | 3.579 | 0.0948 | 4.543 | 0.0629 |
| qanti-TNFa | 27.13 | 0.001998 | 21.9 | 0.001749 | 52.95 | 0.000154 |
| anti-TNFa | 2.936 | 0.137451 | 407.923 | 3.89E-07 | 54.299 | 0.000144 |
| Qvol | 1.995 | 0.20753 | 86.258 | 3.80E-05 | 47.468 | 0.00021 |
| Qglc | 8.435 | 0.0272 | 96.444 | 2.75E-05 | 8.131 | 0.0196 |
| Qlac | 157.87 | 1.56E-05 | 47.86 | 0.000205 | 26.08 | 0.001098 |
| qlac2 | 58.02 | 0.000267 | 35.58 | 0.00047 | 48.6 | 0.000196 |
| Qgln | 0.289 | 0.6101 | 6.477 | 0.0317 | 1.128 | 0.3838 |
| qgln2 | 44.18 | 0.00056 | 20.8 | 0.002 | 14.04 | 0.00545 |
