# Supplementary material for: Mild hypothermia upregulates myc and xbp1s expression and improves anti-TNFα production in CHO cells
Source: PLoS One. 2018 Mar 22;13(3):e0194510. doi: 10.1371/journal.pone.0194510 (PMC5864046; doi:10.1371/journal.pone.0194510)
Supplement: S2 Table — (DOCX) [file pone.0194510.s004.docx]

**S2 Table. Tukey HSD test for the comparison of physiological paramenters between clone type and culture temperature samples.**

| **Parameter** | **Comparison** | **diff** | **lwr** | **upr** | **p adj** |
| --- | --- | --- | --- | --- | --- |
| Xmax | CN2:T31-CN1:T31 | -1.475 | -2.440 | -0.510 | 6.81E-03 |
|  | CN1:T33-CN1:T31 | -0.085 | -1.050 | 0.880 | 9.99E-01 |
|  | CN2:T33-CN1:T31 | -0.930 | -1.895 | 0.035 | 5.83E-02 |
|  | CN1:T37-CN1:T31 | -0.815 | -1.780 | 0.150 | 9.79E-02 |
|  | CN2:T37-CN1:T31 | -1.565 | -2.530 | -0.600 | 5.02E-03 |
|  | CN1:T33-CN2:T31 | 1.390 | 0.425 | 2.355 | 9.19E-03 |
|  | CN2:T33-CN2:T31 | 0.545 | -0.420 | 1.510 | 3.36E-01 |
|  | CN1:T37-CN2:T31 | 0.660 | -0.305 | 1.625 | 2.00E-01 |
|  | CN2:T37-CN2:T31 | -0.090 | -1.055 | 0.875 | 9.99E-01 |
|  | CN2:T33-CN1:T33 | -0.845 | -1.810 | 0.120 | 8.53E-02 |
|  | CN1:T37-CN1:T33 | -0.730 | -1.695 | 0.235 | 1.45E-01 |
|  | CN2:T37-CN1:T33 | -1.480 | -2.445 | -0.515 | 6.69E-03 |
|  | CN1:T37-CN2:T33 | 0.115 | -0.850 | 1.080 | 9.95E-01 |
|  | CN2:T37-CN2:T33 | -0.635 | -1.600 | 0.330 | 2.24E-01 |
|  | CN2:T37-CN1:T37 | -0.750 | -1.715 | 0.215 | 1.32E-01 |
|  | T33-T31 | 0.230 | -0.296 | 0.756 | 4.25E-01 |
|  | T37-T31 | -0.453 | -0.979 | 0.074 | 8.55E-02 |
|  | T37-T33 | -0.683 | -1.209 | -0.156 | 1.71E-02 |
| μ | CN2:T31-CN1:T31 | -0.001 | -0.005 | 0.003 | 9.28E-01 |
|  | CN1:T33-CN1:T31 | 0.003 | -0.001 | 0.007 | 1.63E-01 |
|  | CN2:T33-CN1:T31 | 0.000 | -0.004 | 0.004 | 9.99E-01 |
|  | CN1:T37-CN1:T31 | 0.002 | -0.002 | 0.006 | 3.81E-01 |
|  | CN2:T37-CN1:T31 | -0.001 | -0.005 | 0.003 | 8.40E-01 |
|  | CN1:T33-CN2:T31 | 0.004 | 0.000 | 0.008 | 5.92E-02 |
|  | CN2:T33-CN2:T31 | 0.001 | -0.003 | 0.005 | 7.97E-01 |
|  | CN1:T37-CN2:T31 | 0.003 | -0.001 | 0.007 | 1.39E-01 |
|  | CN2:T37-CN2:T31 | 0.000 | -0.004 | 0.004 | 1.00E+00 |
|  | CN2:T33-CN1:T33 | -0.003 | -0.007 | 0.001 | 2.39E-01 |
|  | CN1:T37-CN1:T33 | -0.001 | -0.005 | 0.003 | 9.63E-01 |
|  | CN2:T37-CN1:T33 | -0.004 | -0.008 | 0.000 | 4.58E-02 |
|  | CN1:T37-CN2:T33 | 0.002 | -0.002 | 0.006 | 5.31E-01 |
|  | CN2:T37-CN2:T33 | -0.002 | -0.006 | 0.003 | 6.77E-01 |
|  | CN2:T37-CN1:T37 | -0.003 | -0.008 | 0.001 | 1.06E-01 |
|  | T33-T31 | 0.002 | 0.000 | 0.004 | 5.85E-02 |
|  | T37-T31 | 0.001 | -0.001 | 0.003 | 4.29E-01 |
|  | T37-T33 | -0.001 | -0.003 | 0.001 | 3.13E-01 |
| qanti-TNFa | CN2:T31-CN1:T31 | 254.400 | 151.438 | 357.362 | 5.09E-04 |
|  | CN1:T33-CN1:T31 | 8.250 | -94.712 | 111.212 | 9.99E-01 |
|  | CN2:T33-CN1:T31 | 107.500 | 4.538 | 210.462 | 4.16E-02 |
|  | CN1:T37-CN1:T31 | 66.700 | -36.262 | 169.662 | 2.35E-01 |
|  | CN2:T37-CN1:T31 | -53.550 | -156.512 | 49.412 | 4.05E-01 |
|  | CN1:T33-CN2:T31 | -246.150 | -349.112 | -143.188 | 6.13E-04 |
|  | CN2:T33-CN2:T31 | -146.900 | -249.862 | -43.938 | 9.65E-03 |
|  | CN1:T37-CN2:T31 | -187.700 | -290.662 | -84.738 | 2.71E-03 |
|  | CN2:T37-CN2:T31 | -307.950 | -410.912 | -204.988 | 1.73E-04 |
|  | CN2:T33-CN1:T33 | 99.250 | -3.712 | 202.212 | 5.83E-02 |
|  | CN1:T37-CN1:T33 | 58.450 | -44.512 | 161.412 | 3.32E-01 |
|  | CN2:T37-CN1:T33 | -61.800 | -164.762 | 41.162 | 2.89E-01 |
|  | CN1:T37-CN2:T33 | -40.800 | -143.762 | 62.162 | 6.38E-01 |
|  | CN2:T37-CN2:T33 | -161.050 | -264.012 | -58.088 | 6.05E-03 |
|  | CN2:T37-CN1:T37 | -120.250 | -223.212 | -17.288 | 2.52E-02 |
|  | T33-T31 | -69.325 | -125.454 | -13.196 | 2.12E-02 |
|  | T37-T31 | -120.625 | -176.754 | -64.496 | 1.42E-03 |
|  | T37-T33 | -51.300 | -107.429 | 4.829 | 6.94E-02 |
| qglc | CN2:T31-CN1:T31 | -5.550 | -29.957 | 18.857 | 9.32E-01 |
|  | CN1:T33-CN1:T31 | -5.950 | -30.357 | 18.457 | 9.12E-01 |
|  | CN2:T33-CN1:T31 | -1.600 | -26.007 | 22.807 | 1.00E+00 |
|  | CN1:T37-CN1:T31 | -40.600 | -65.007 | -16.193 | 4.40E-03 |
|  | CN2:T37-CN1:T31 | -70.250 | -94.657 | -45.843 | 2.15E-04 |
|  | CN1:T33-CN2:T31 | -0.400 | -24.807 | 24.007 | 1.00E+00 |
|  | CN2:T33-CN2:T31 | 3.950 | -20.457 | 28.357 | 9.82E-01 |
|  | CN1:T37-CN2:T31 | -35.050 | -59.457 | -10.643 | 9.34E-03 |
|  | CN2:T37-CN2:T31 | -64.700 | -89.107 | -40.293 | 3.42E-04 |
|  | CN2:T33-CN1:T33 | 4.350 | -20.057 | 28.757 | 9.74E-01 |
|  | CN1:T37-CN1:T33 | -34.650 | -59.057 | -10.243 | 9.89E-03 |
|  | CN2:T37-CN1:T33 | -64.300 | -88.707 | -39.893 | 3.55E-04 |
|  | CN1:T37-CN2:T33 | -39.000 | -63.407 | -14.593 | 5.42E-03 |
|  | CN2:T37-CN2:T33 | -68.650 | -93.057 | -44.243 | 2.45E-04 |
|  | CN2:T37-CN1:T37 | -29.650 | -54.057 | -5.243 | 2.10E-02 |
|  | T33-T31 | -1.000 | -14.305 | 12.305 | 9.71E-01 |
|  | T37-T31 | -52.650 | -65.955 | -39.345 | 4.68E-05 |
|  | T37-T33 | -51.650 | -64.955 | -38.345 | 5.23E-05 |
| qgln | CN2:T31-CN1:T31 | 3.050 | -4.856 | 10.956 | 6.59E-01 |
|  | CN1:T33-CN1:T31 | -3.000 | -10.906 | 4.906 | 6.72E-01 |
|  | CN2:T33-CN1:T31 | -3.500 | -11.406 | 4.406 | 5.45E-01 |
|  | CN1:T37-CN1:T31 | -1.950 | -9.856 | 5.956 | 9.09E-01 |
|  | CN2:T37-CN1:T31 | -2.650 | -10.556 | 5.256 | 7.61E-01 |
|  | CN1:T33-CN2:T31 | -6.050 | -13.956 | 1.856 | 1.39E-01 |
|  | CN2:T33-CN2:T31 | -6.550 | -14.456 | 1.356 | 1.05E-01 |
|  | CN1:T37-CN2:T31 | -5.000 | -12.906 | 2.906 | 2.51E-01 |
|  | CN2:T37-CN2:T31 | -5.700 | -13.606 | 2.206 | 1.70E-01 |
|  | CN2:T33-CN1:T33 | -0.500 | -8.406 | 7.406 | 1.00E+00 |
|  | CN1:T37-CN1:T33 | 1.050 | -6.856 | 8.956 | 9.93E-01 |
|  | CN2:T37-CN1:T33 | 0.350 | -7.556 | 8.256 | 1.00E+00 |
|  | CN1:T37-CN2:T33 | 1.550 | -6.356 | 9.456 | 9.61E-01 |
|  | CN2:T37-CN2:T33 | 0.850 | -7.056 | 8.756 | 9.97E-01 |
|  | CN2:T37-CN1:T37 | -0.700 | -8.606 | 7.206 | 9.99E-01 |
|  | T33-T31 | -4.775 | -9.085 | -0.465 | 3.35E-02 |
|  | T37-T31 | -3.825 | -8.135 | 0.485 | 7.69E-02 |
|  | T37-T33 | 0.950 | -3.360 | 5.260 | 7.85E-01 |
| qgln2 | CN2:T31-CN1:T31 | 11.350 | 5.318 | 17.382 | 2.29E-03 |
|  | CN1:T33-CN1:T31 | 0.650 | -5.382 | 6.682 | 9.97E-01 |
|  | CN2:T33-CN1:T31 | 6.750 | 0.718 | 12.782 | 3.06E-02 |
|  | CN1:T37-CN1:T31 | -1.050 | -7.082 | 4.982 | 9.76E-01 |
|  | CN2:T37-CN1:T31 | -1.050 | -7.082 | 4.982 | 9.76E-01 |
|  | CN1:T33-CN2:T31 | -10.700 | -16.732 | -4.668 | 3.14E-03 |
|  | CN2:T33-CN2:T31 | -4.600 | -10.632 | 1.432 | 1.41E-01 |
|  | CN1:T37-CN2:T31 | -12.400 | -18.432 | -6.368 | 1.42E-03 |
|  | CN2:T37-CN2:T31 | -12.400 | -18.432 | -6.368 | 1.42E-03 |
|  | CN2:T33-CN1:T33 | 6.100 | 0.068 | 12.132 | 4.77E-02 |
|  | CN1:T37-CN1:T33 | -1.700 | -7.732 | 4.332 | 8.57E-01 |
|  | CN2:T37-CN1:T33 | -1.700 | -7.732 | 4.332 | 8.57E-01 |
|  | CN1:T37-CN2:T33 | -7.800 | -13.832 | -1.768 | 1.56E-02 |
|  | CN2:T37-CN2:T33 | -7.800 | -13.832 | -1.768 | 1.56E-02 |
|  | CN2:T37-CN1:T37 | 0.000 | -6.032 | 6.032 | 1.00E+00 |
|  | T33-T31 | -1.975 | -5.264 | 1.314 | 2.35E-01 |
|  | T37-T31 | -6.725 | -10.014 | -3.436 | 1.85E-03 |
|  | T37-T33 | -4.750 | -8.039 | -1.461 | 1.05E-02 |
| qlac | CN2:T31-CN1:T31 | -53.500 | -77.393 | -29.607 | 8.83E-04 |
|  | CN1:T33-CN1:T31 | 4.700 | -19.193 | 28.593 | 9.61E-01 |
|  | CN2:T33-CN1:T31 | -63.300 | -87.193 | -39.407 | 3.43E-04 |
|  | CN1:T37-CN1:T31 | 12.450 | -11.443 | 36.343 | 4.03E-01 |
|  | CN2:T37-CN1:T31 | 3.300 | -20.593 | 27.193 | 9.91E-01 |
|  | CN1:T33-CN2:T31 | 58.200 | 34.307 | 82.093 | 5.52E-04 |
|  | CN2:T33-CN2:T31 | -9.800 | -33.693 | 14.093 | 6.10E-01 |
|  | CN1:T37-CN2:T31 | 65.950 | 42.057 | 89.843 | 2.72E-04 |
|  | CN2:T37-CN2:T31 | 56.800 | 32.907 | 80.693 | 6.32E-04 |
|  | CN2:T33-CN1:T33 | -68.000 | -91.893 | -44.107 | 2.29E-04 |
|  | CN1:T37-CN1:T33 | 7.750 | -16.143 | 31.643 | 7.82E-01 |
|  | CN2:T37-CN1:T33 | -1.400 | -25.293 | 22.493 | 1.00E+00 |
|  | CN1:T37-CN2:T33 | 75.750 | 51.857 | 99.643 | 1.24E-04 |
|  | CN2:T37-CN2:T33 | 66.600 | 42.707 | 90.493 | 2.58E-04 |
|  | CN2:T37-CN1:T37 | -9.150 | -33.043 | 14.743 | 6.65E-01 |
|  | T33-T31 | -2.550 | -15.575 | 10.475 | 8.25E-01 |
|  | T37-T31 | 34.625 | 21.600 | 47.650 | 4.48E-04 |
|  | T37-T33 | 37.175 | 24.150 | 50.200 | 3.02E-04 |
| qlac2 | CN2:T31-CN1:T31 | -34.750 | -49.365 | -20.135 | 6.32E-04 |
|  | CN1:T33-CN1:T31 | 8.250 | -6.365 | 22.865 | 3.37E-01 |
|  | CN2:T33-CN1:T31 | -18.500 | -33.115 | -3.885 | 1.73E-02 |
|  | CN1:T37-CN1:T31 | -2.050 | -16.665 | 12.565 | 9.91E-01 |
|  | CN2:T37-CN1:T31 | 11.000 | -3.615 | 25.615 | 1.47E-01 |
|  | CN1:T33-CN2:T31 | 43.000 | 28.385 | 57.615 | 1.89E-04 |
|  | CN2:T33-CN2:T31 | 16.250 | 1.635 | 30.865 | 3.15E-02 |
|  | CN1:T37-CN2:T31 | 32.700 | 18.085 | 47.315 | 8.87E-04 |
|  | CN2:T37-CN2:T31 | 45.750 | 31.135 | 60.365 | 1.33E-04 |
|  | CN2:T33-CN1:T33 | -26.750 | -41.365 | -12.135 | 2.65E-03 |
|  | CN1:T37-CN1:T33 | -10.300 | -24.915 | 4.315 | 1.82E-01 |
|  | CN2:T37-CN1:T33 | 2.750 | -11.865 | 17.365 | 9.67E-01 |
|  | CN1:T37-CN2:T33 | 16.450 | 1.835 | 31.065 | 2.98E-02 |
|  | CN2:T37-CN2:T33 | 29.500 | 14.885 | 44.115 | 1.56E-03 |
|  | CN2:T37-CN1:T37 | 13.050 | -1.565 | 27.665 | 7.92E-02 |
|  | T33-T31 | 12.250 | 4.283 | 20.217 | 7.79E-03 |
|  | T37-T31 | 21.850 | 13.883 | 29.817 | 3.77E-04 |
|  | T37-T33 | 9.600 | 1.633 | 17.567 | 2.36E-02 |
| Qvol | CN2:T31-CN1:T31 | 0.091 | 0.011 | 0.171 | 2.91E-02 |
|  | CN1:T33-CN1:T31 | -0.055 | -0.136 | 0.025 | 1.94E-01 |
|  | CN2:T33-CN1:T31 | 0.047 | -0.033 | 0.127 | 3.09E-01 |
|  | CN1:T37-CN1:T31 | -0.064 | -0.144 | 0.016 | 1.21E-01 |
|  | CN2:T37-CN1:T31 | -0.208 | -0.288 | -0.127 | 3.92E-04 |
|  | CN1:T33-CN2:T31 | -0.146 | -0.226 | -0.066 | 2.72E-03 |
|  | CN2:T33-CN2:T31 | -0.044 | -0.124 | 0.036 | 3.63E-01 |
|  | CN1:T37-CN2:T31 | -0.155 | -0.235 | -0.074 | 2.01E-03 |
|  | CN2:T37-CN2:T31 | -0.298 | -0.379 | -0.218 | 4.87E-05 |
|  | CN2:T33-CN1:T33 | 0.102 | 0.022 | 0.183 | 1.67E-02 |
|  | CN1:T37-CN1:T33 | -0.008 | -0.089 | 0.072 | 9.97E-01 |
|  | CN2:T37-CN1:T33 | -0.152 | -0.232 | -0.072 | 2.18E-03 |
|  | CN1:T37-CN2:T33 | -0.111 | -0.191 | -0.031 | 1.13E-02 |
|  | CN2:T37-CN2:T33 | -0.255 | -0.335 | -0.174 | 1.23E-04 |
|  | CN2:T37-CN1:T37 | -0.144 | -0.224 | -0.064 | 2.97E-03 |
|  | T33-T31 | -0.050 | -0.093 | -0.006 | 3.03E-02 |
|  | T37-T31 | -0.181 | -0.225 | -0.137 | 3.59E-05 |
|  | T37-T33 | -0.131 | -0.175 | -0.088 | 2.25E-04 |
| Ylac | CN2:T31-CN1:T31 | 0.091 | 0.011 | 0.171 | 2.91E-02 |
|  | CN1:T33-CN1:T31 | -0.055 | -0.136 | 0.025 | 1.94E-01 |
|  | CN2:T33-CN1:T31 | 0.047 | -0.033 | 0.127 | 3.09E-01 |
|  | CN1:T37-CN1:T31 | -0.064 | -0.144 | 0.016 | 1.21E-01 |
|  | CN2:T37-CN1:T31 | -0.208 | -0.288 | -0.127 | 3.92E-04 |
|  | CN1:T33-CN2:T31 | -0.146 | -0.226 | -0.066 | 2.72E-03 |
|  | CN2:T33-CN2:T31 | -0.044 | -0.124 | 0.036 | 3.63E-01 |
|  | CN1:T37-CN2:T31 | -0.155 | -0.235 | -0.074 | 2.01E-03 |
|  | CN2:T37-CN2:T31 | -0.298 | -0.379 | -0.218 | 4.87E-05 |
|  | CN2:T33-CN1:T33 | 0.102 | 0.022 | 0.183 | 1.67E-02 |
|  | CN1:T37-CN1:T33 | -0.008 | -0.089 | 0.072 | 9.97E-01 |
|  | CN2:T37-CN1:T33 | -0.152 | -0.232 | -0.072 | 2.18E-03 |
|  | CN1:T37-CN2:T33 | -0.111 | -0.191 | -0.031 | 1.13E-02 |
|  | CN2:T37-CN2:T33 | -0.255 | -0.335 | -0.174 | 1.23E-04 |
|  | CN2:T37-CN1:T37 | -0.144 | -0.224 | -0.064 | 2.97E-03 |
|  | T33-T31 | -0.050 | -0.093 | -0.006 | 3.03E-02 |
|  | T37-T31 | -0.181 | -0.225 | -0.137 | 3.59E-05 |
|  | T37-T33 | -0.131 | -0.175 | -0.088 | 2.25E-04 |
