# Supplementary material for: Mild hypothermia upregulates myc and xbp1s expression and improves anti-TNFα production in CHO cells
Source: PLoS One. 2018 Mar 22;13(3):e0194510. doi: 10.1371/journal.pone.0194510 (PMC5864046; doi:10.1371/journal.pone.0194510)
Supplement: S3 Table — (DOCX) [file pone.0194510.s005.docx]

S3 Table. Impact of clone type and culture temperature on the differential expressions of mRNA encoding for anti-TNFα, Myc and XBP1S at 6 and 72h (Two-way ANOVA factors; n=3).

|  |  | **Clone** | | **Temperature** | | **Clone:Temperature** | |
| --- | --- | --- | --- | --- | --- | --- | --- |
|  |  | **F value** | **P value** | **F value** | **P value** | **F value** | **P value** |
| 6 h | Myc | 5.131 | 0.0641 | 10.844 | 0.0102 | 1.121 | 0.3859 |
|  | xbp1 | 243.4 | 4.39E-06 | 288.9 | 1.09E-06 | 258.1 | 1.52E-06 |
|  | anti-TNFa | 950.4 | 7.73E-08 | 901.5 | 3.65E-08 | 814.2 | 4.95E-08 |
| 72 h | myc | 7404 | 1.66E-10 | 9243 | 3.42E-11 | 5874 | 1.33E-10 |
|  | xbp1 | 144.1 | 2.03E-05 | 29.8 | 0.000765 | 36.99 | 4.22E-04 |
|  | anti-TNFa | 959.4 | 7.52E-08 | 915.3 | 3.49E-08 | 901.30 | 3.65E-08 |
