# Supplementary material for: Mild hypothermia upregulates myc and xbp1s expression and improves anti-TNFα production in CHO cells
Source: PLoS One. 2018 Mar 22;13(3):e0194510. doi: 10.1371/journal.pone.0194510 (PMC5864046; doi:10.1371/journal.pone.0194510)
Supplement: S4 Table — (DOCX) [file pone.0194510.s006.docx]

**S4 Table. T-test of the differential expressions of mRNA encoding for anti-TNFα, Myc and XBP1S between 6 and 72h in CN1 and CN2 at 37, 33 and 31°C.**

|  |  | **37°C** |  | **33°C** |  | **37°C** |  |
| --- | --- | --- | --- | --- | --- | --- | --- |
|  |  | t-value | p-value | t-value | p-value | t-value | p-value |
| **myc** | **CN1** | -9.06842105 | 0.03495967 | -12.4764675 | 0.0254584 | -30.3996992 | 0.01046705 |
|  | **CN2** | -1.11750881 | 0.23235384 | -14.9577889 | 0.02124892 | -13.027972 | 0.02438499 |
| **xbp1** | **CN1** | -2.40696279 | 0.12533887 | -2.81387766 | 0.10869039 | -1.82264824 | 0.1597304 |
|  | **CN2** | -6.00009628 | 0.05025685 | -19.542158 | 0.01627417 | -49.3544304 | 0.00644859 |
| **anti-TNFα** | **CN1** | -11.0876866 | 0.02863095 | -4.61008933 | 0.06799301 | -8.65518257 | 0.03661445 |
|  | **CN2** | -10.913691 | 0.02908489 | -110.113182 | 0.00289067 | -30.5133259 | 0.0104281 |
