# Supplementary material for: Mild hypothermia upregulates myc and xbp1s expression and improves anti-TNFα production in CHO cells
Source: PLoS One. 2018 Mar 22;13(3):e0194510. doi: 10.1371/journal.pone.0194510 (PMC5864046; doi:10.1371/journal.pone.0194510)
Supplement: S5 Table — (DOCX) [file pone.0194510.s007.docx]

S5 Table. Tukey HSD test for the comparison of the differential expressions of mRNA encoding for anti-TNFα, Myc and XBP1S at 6 and 72h between clone type and culture temperature samples.

| **Gene** | **Time** | **Comparison** | **diff** | **lwr** | **upr** | **p adj** |
| --- | --- | --- | --- | --- | --- | --- |
| anti-TNFα | 6 h | CN2:X31-CN1:X31 | 22.746 | 20.962 | 24.531 | 1.54E-08 |
|  |  | CN1:X33-CN1:X31 | -0.606 | -2.390 | 1.179 | 7.53E-01 |
|  |  | CN2:X33-CN1:X31 | 0.428 | -1.357 | 2.212 | 9.17E-01 |
|  |  | CN1:X37-CN1:X31 | -0.556 | -2.341 | 1.228 | 8.05E-01 |
|  |  | CN2:X37-CN1:X31 | -0.393 | -2.178 | 1.391 | 9.39E-01 |
|  |  | CN1:X33-CN2:X31 | -23.352 | -25.137 | -21.567 | 1.05E-08 |
|  |  | CN2:X33-CN2:X31 | -22.319 | -24.103 | -20.534 | 2.00E-08 |
|  |  | CN1:X37-CN2:X31 | -23.303 | -25.087 | -21.518 | 1.09E-08 |
|  |  | CN2:X37-CN2:X31 | -23.140 | -24.924 | -21.355 | 1.20E-08 |
|  |  | CN2:X33-CN1:X33 | 1.033 | -0.751 | 2.818 | 3.17E-01 |
|  |  | CN1:X37-CN1:X33 | 0.049 | -1.735 | 1.834 | 1.00E+00 |
|  |  | CN2:X37-CN1:X33 | 0.212 | -1.572 | 1.997 | 9.95E-01 |
|  |  | CN1:X37-CN2:X33 | -0.984 | -2.768 | 0.801 | 3.56E-01 |
|  |  | CN2:X37-CN2:X33 | -0.821 | -2.605 | 0.964 | 5.11E-01 |
|  |  | CN2:X37-CN1:X37 | 0.163 | -1.622 | 1.948 | 9.99E-01 |
|  |  | X33-X31 | -11.462 | -12.435 | -10.489 | 1.17E-07 |
|  |  | X37-X31 | -11.848 | -12.821 | -10.875 | 9.08E-08 |
|  |  | X37-X33 | -0.386 | -1.359 | 0.587 | 4.87E-01 |
|  | 72 h | CN2:X31-CN1:X31 | 277.074 | 256.089 | 298.058 | 9.20E-09 |
|  |  | CN1:X33-CN1:X31 | -0.769 | -21.753 | 20.216 | 1.00E+00 |
|  |  | CN2:X33-CN1:X31 | 3.522 | -17.463 | 24.507 | 9.80E-01 |
|  |  | CN1:X37-CN1:X31 | -1.338 | -22.323 | 19.646 | 1.00E+00 |
|  |  | CN2:X37-CN1:X31 | 0.179 | -20.806 | 21.164 | 1.00E+00 |
|  |  | CN1:X33-CN2:X31 | -277.842 | -298.827 | -256.858 | 8.82E-09 |
|  |  | CN2:X33-CN2:X31 | -273.552 | -294.536 | -252.567 | 1.11E-08 |
|  |  | CN1:X37-CN2:X31 | -278.412 | -299.397 | -257.427 | 8.55E-09 |
|  |  | CN2:X37-CN2:X31 | -276.895 | -297.880 | -255.910 | 9.29E-09 |
|  |  | CN2:X33-CN1:X33 | 4.291 | -16.694 | 25.275 | 9.54E-01 |
|  |  | CN1:X37-CN1:X33 | -0.570 | -21.554 | 20.415 | 1.00E+00 |
|  |  | CN2:X37-CN1:X33 | 0.947 | -20.037 | 21.932 | 1.00E+00 |
|  |  | CN1:X37-CN2:X33 | -4.860 | -25.845 | 16.124 | 9.27E-01 |
|  |  | CN2:X37-CN2:X33 | -3.343 | -24.328 | 17.641 | 9.84E-01 |
|  |  | CN2:X37-CN1:X37 | 1.517 | -19.468 | 22.502 | 1.00E+00 |
|  |  | X33-X31 | -137.160 | -148.600 | -125.720 | 1.03E-07 |
|  |  | X37-X31 | -139.117 | -150.556 | -127.677 | 9.18E-08 |
|  |  | X37-X33 | -1.957 | -13.396 | 9.483 | 8.62E-01 |
| *myc* | 6 h | CN2:X31-CN1:X31 | 0.052 | -0.444 | 0.548 | 9.98E-01 |
|  |  | CN1:X33-CN1:X31 | -0.207 | -0.703 | 0.289 | 5.94E-01 |
|  |  | CN2:X33-CN1:X31 | 0.102 | -0.394 | 0.598 | 9.54E-01 |
|  |  | CN1:X37-CN1:X31 | -0.427 | -0.923 | 0.069 | 9.13E-02 |
|  |  | CN2:X37-CN1:X31 | -0.298 | -0.794 | 0.198 | 2.88E-01 |
|  |  | CN1:X33-CN2:X31 | -0.259 | -0.755 | 0.237 | 4.02E-01 |
|  |  | CN2:X33-CN2:X31 | 0.050 | -0.446 | 0.546 | 9.98E-01 |
|  |  | CN1:X37-CN2:X31 | -0.478 | -0.974 | 0.018 | 5.82E-02 |
|  |  | CN2:X37-CN2:X31 | -0.350 | -0.846 | 0.146 | 1.82E-01 |
|  |  | CN2:X33-CN1:X33 | 0.309 | -0.187 | 0.805 | 2.63E-01 |
|  |  | CN1:X37-CN1:X33 | -0.220 | -0.716 | 0.276 | 5.45E-01 |
|  |  | CN2:X37-CN1:X33 | -0.091 | -0.587 | 0.405 | 9.71E-01 |
|  |  | CN1:X37-CN2:X33 | -0.528 | -1.024 | -0.032 | 3.81E-02 |
|  |  | CN2:X37-CN2:X33 | -0.400 | -0.896 | 0.096 | 1.16E-01 |
|  |  | CN2:X37-CN1:X37 | 0.129 | -0.367 | 0.625 | 8.91E-01 |
|  |  | X33-X31 | -0.079 | -0.349 | 0.192 | 6.65E-01 |
|  |  | X37-X31 | -0.388 | -0.659 | -0.118 | 1.08E-02 |
|  |  | X37-X33 | -0.310 | -0.580 | -0.039 | 2.93E-02 |
|  | 72 h | CN2:X31-CN1:X31 | -2.346 | -2.414 | -2.278 | 6.42E-14 |
|  |  | CN1:X33-CN1:X31 | -2.144 | -2.212 | -2.077 | 6.42E-14 |
|  |  | CN2:X33-CN1:X31 | -2.337 | -2.405 | -2.270 | 6.42E-14 |
|  |  | CN1:X37-CN1:X31 | -2.781 | -2.849 | -2.714 | 6.42E-14 |
|  |  | CN2:X37-CN1:X31 | -2.777 | -2.845 | -2.710 | 6.42E-14 |
|  |  | CN1:X33-CN2:X31 | 0.202 | 0.134 | 0.269 | 1.77E-04 |
|  |  | CN2:X33-CN2:X31 | 0.009 | -0.059 | 0.076 | 9.94E-01 |
|  |  | CN1:X37-CN2:X31 | -0.435 | -0.503 | -0.368 | 2.24E-06 |
|  |  | CN2:X37-CN2:X31 | -0.431 | -0.499 | -0.364 | 2.31E-06 |
|  |  | CN2:X33-CN1:X33 | -0.193 | -0.261 | -0.125 | 2.26E-04 |
|  |  | CN1:X37-CN1:X33 | -0.637 | -0.705 | -0.569 | 3.55E-07 |
|  |  | CN2:X37-CN1:X33 | -0.633 | -0.701 | -0.565 | 3.71E-07 |
|  |  | CN1:X37-CN2:X33 | -0.444 | -0.512 | -0.376 | 2.10E-06 |
|  |  | CN2:X37-CN2:X33 | -0.440 | -0.508 | -0.372 | 2.16E-06 |
|  |  | CN2:X37-CN1:X37 | 0.004 | -0.064 | 0.072 | 1.00E+00 |
|  |  | X33-X31 | -1.068 | -1.105 | -1.031 | 6.57E-14 |
|  |  | X37-X31 | -1.606 | -1.643 | -1.570 | 6.42E-14 |
|  |  | X37-X33 | -0.539 | -0.575 | -0.502 | 1.62E-08 |
| *xbp1* | 6 h | CN2:X31-CN1:X31 | 0.497 | 0.425 | 0.568 | 1.73E-06 |
|  |  | CN1:X33-CN1:X31 | -0.008 | -0.079 | 0.064 | 9.97E-01 |
|  |  | CN2:X33-CN1:X31 | -0.009 | -0.081 | 0.063 | 9.94E-01 |
|  |  | CN1:X37-CN1:X31 | -0.021 | -0.092 | 0.051 | 8.48E-01 |
|  |  | CN2:X37-CN1:X31 | -0.029 | -0.101 | 0.043 | 6.23E-01 |
|  |  | CN1:X33-CN2:X31 | -0.504 | -0.576 | -0.433 | 1.64E-06 |
|  |  | CN2:X33-CN2:X31 | -0.506 | -0.577 | -0.434 | 1.62E-06 |
|  |  | CN1:X37-CN2:X31 | -0.517 | -0.589 | -0.445 | 1.49E-06 |
|  |  | CN2:X37-CN2:X31 | -0.526 | -0.597 | -0.454 | 1.40E-06 |
|  |  | CN2:X33-CN1:X33 | -0.001 | -0.073 | 0.070 | 1.00E+00 |
|  |  | CN1:X37-CN1:X33 | -0.013 | -0.085 | 0.059 | 9.73E-01 |
|  |  | CN2:X37-CN1:X33 | -0.021 | -0.093 | 0.050 | 8.33E-01 |
|  |  | CN1:X37-CN2:X33 | -0.012 | -0.083 | 0.060 | 9.82E-01 |
|  |  | CN2:X37-CN2:X33 | -0.020 | -0.092 | 0.052 | 8.63E-01 |
|  |  | CN2:X37-CN1:X37 | -0.008 | -0.080 | 0.063 | 9.96E-01 |
|  |  | X33-X31 | -0.257 | -0.296 | -0.218 | 2.24E-06 |
|  |  | X37-X31 | -0.273 | -0.312 | -0.234 | 1.64E-06 |
|  |  | X37-X33 | -0.016 | -0.056 | 0.023 | 4.51E-01 |
|  | 72 h | CN2:X31-CN1:X31 | 0.664 | 0.474 | 0.854 | 7.04E-05 |
|  |  | CN1:X33-CN1:X31 | -0.002 | -0.192 | 0.188 | 1.00E+00 |
|  |  | CN2:X33-CN1:X31 | 0.193 | 0.003 | 0.383 | 4.65E-02 |
|  |  | CN1:X37-CN1:X31 | 0.052 | -0.138 | 0.242 | 8.67E-01 |
|  |  | CN2:X37-CN1:X31 | 0.186 | -0.004 | 0.376 | 5.52E-02 |
|  |  | CN1:X33-CN2:X31 | -0.666 | -0.856 | -0.476 | 6.92E-05 |
|  |  | CN2:X33-CN2:X31 | -0.471 | -0.661 | -0.281 | 5.00E-04 |
|  |  | CN1:X37-CN2:X31 | -0.612 | -0.802 | -0.422 | 1.13E-04 |
|  |  | CN2:X37-CN2:X31 | -0.479 | -0.669 | -0.289 | 4.56E-04 |
|  |  | CN2:X33-CN1:X33 | 0.195 | 0.005 | 0.385 | 4.46E-02 |
|  |  | CN1:X37-CN1:X33 | 0.054 | -0.136 | 0.244 | 8.50E-01 |
|  |  | CN2:X37-CN1:X33 | 0.187 | -0.003 | 0.378 | 5.29E-02 |
|  |  | CN1:X37-CN2:X33 | -0.141 | -0.331 | 0.049 | 1.55E-01 |
|  |  | CN2:X37-CN2:X33 | -0.008 | -0.198 | 0.182 | 1.00E+00 |
|  |  | CN2:X37-CN1:X37 | 0.133 | -0.057 | 0.323 | 1.86E-01 |
|  |  | X33-X31 | -0.236 | -0.340 | -0.133 | 1.03E-03 |
|  |  | X37-X31 | -0.213 | -0.317 | -0.110 | 1.79E-03 |
|  |  | X37-X33 | 0.023 | -0.080 | 0.127 | 7.78E-01 |
